# Regulation of the *THRA* gene, encoding the thyroid hormone nuclear receptor TRα1, in intestinal lesions

**DOI:** 10.1002/1878-0261.13298

**Published:** 2022-10-10

**Authors:** Maria Virginia Giolito, Théo La Rosa, Diana Farhat, Serguei Bodoirat, Gabriela D. A. Guardia, Claire Domon‐Dell, Pedro A. F. Galante, Jean‐Noel Freund, Michelina Plateroti

**Affiliations:** ^1^ Inserm, IRFAC/UMR‐S1113, FMTS, Université de Strasbourg France; ^2^ INSERM U1052, CNRS UMR5286, Centre de Recherche en Cancérologie de Lyon France; ^3^ Centro de Oncologia Molecular Hospital Sírio‐Libanês São Paulo Brazil; ^4^ Present address: Stem‐Cell and Brain Research Institute, U1208 INSERM, USC1361 INRA Bron France

**Keywords:** colon cancer, intestinal organoids, THRA, thyroid hormone nuclear receptor, TRα1

## Abstract

The *THRA* gene, encoding the thyroid hormone nuclear receptor TRα1, is expressed in an increasing gradient at the bottom of intestinal crypts, overlapping with high Wnt and Notch activities. Importantly, *THRA* is upregulated in colorectal cancers, particularly in the high‐Wnt molecular subtype. The basis of this specific and/or altered expression pattern has remained unknown. To define the mechanisms controlling *THRA* transcription and TRα1 expression, we used multiple *in vitro* and *ex vivo* approaches. Promoter analysis demonstrated that transcription factors important for crypt homeostasis and altered in colorectal cancers, such as transcription factor 7‐like 2 (TCF7L2; Wnt pathway), recombining binding protein suppressor of hairless (RBPJ; Notch pathway), and homeobox protein CDX2 (epithelial cell identity), modulate *THRA* activity. Specifically, although TCF7L2 and CDX2 stimulated *THRA*, RBPJ induced its repression. In‐depth analysis of the Wnt‐dependent increase showed direct regulation of the *THRA* promoter in cells and of TRα1 expression in murine enteroids. Given our previous results on the control of the Wnt pathway by TRα1, our new results unveil a complex regulatory loop and synergy between these endocrine and epithelial‐cell‐intrinsic signals. Our work describes, for the first time, the regulation of the *THRA* gene in specific cell and tumor contexts.

## Graphical abstract

This study demonstrates the Wnt/β‐catenin‐dependent control of the *THRA* gene, encoding the thyroid hormone nuclear receptor TRα1, in normal colon and colon tumors. *THRA* gene transcription and TRα1 expression are directly regulated by the β‐catenin/TCF complex. The induction of the complex upon *APC* mutation (Wnt‐up) increases TRα1 expression in cancers.

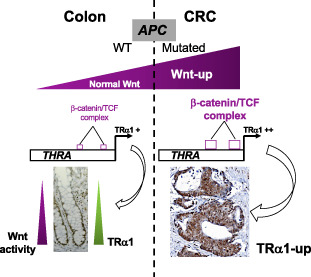

**Abbreviations**
CMS: consensus molecular subtype
CRC: colorectal cancer
IHC: immunohistochemistry
KO: knock‐out
qPCR: quantitative polymerase chain reaction
RLU: relative luciferase units
RT: retro transcription
SC: stem cell
TH: thyroid hormone
THRA: thyroid hormone receptor alpha gene
TMA: tissue microarray analysis
TR: thyroid hormone receptor
WB: western blot

## 1 Introduction

The thyroid hormone (TH) nuclear receptor TRs are T3‐modulated transcription factors belonging to the nuclear hormone receptor protein superfamily [1]. THs and TRs are involved in multiple processes in organism development, physiology, and, eventually, pathological events [2, 3, 4, 5]. From a molecular point of view, they modulate the expression of target genes by binding to thyroid hormone response elements (TREs) present in regulatory regions of target genes. Upon T3 binding, TRs undergo conformational modification, resulting in activation or repression of the transcriptional machinery [2].

One well‐defined organ target of THs and the receptor TRα1 is the intestine. Indeed, the involvement of TRα1‐dependent signaling and/or TH status has been reported in the normal intestine [6, 7, 8, 9] and in intestinal tumor biology [6, 7, 10, 11, 12]. Studies in *Thra*‐ and *Thrb*‐knockout animals showed that TRα1 is responsible for TH signaling in intestinal crypts, where it controls the biology of stem cells (SCs) and their fate [13], as well as the balance between cell proliferation and cell differentiation through its actions on the Wnt and Notch pathways (rev in Refs [6, 7, 10]). In accordance with this important role, overexpression of TRα1 in the intestinal epithelium (*vil*‐TRα1 mice) in a mutated‐Apc background (*vil*‐TRα1/Apc^+/1638N^ mice) is responsible for the acceleration of tumor appearance, progression, and aggressiveness compared with Apc‐only mutants [14]. Conversely, *Thra* gene loss in the same mutated‐Apc background diminishes and slows tumor appearance [15]. Interestingly, the relevance of these observations has been demonstrated in clinics, given that the *THRA* gene and the TRα1 isoform are frequently overexpressed in human colorectal cancer (CRC) patients [15].

CRC is the third leading cause of cancer death in the world [16]. CRC development is a multistep process triggered by the accumulation of mutations in oncogenes and tumor suppressors, which, in turn, are responsible for tumor initiation and progression [17]. Crypt hyperplasia, hypertrophy, and stem cell (SC) transformation represent very early events in intestinal tumor development [18, 19, 20] and depend on alterations of genes in the Wnt and Notch pathways [21, 22]. It is worth noting that these cellular and molecular processes are also affected by TRα1, which synergizes with the Wnt pathway to accelerate neoplastic events [14, 15]. Moreover, the expression of the *THRA* gene is upregulated in CRC consensus molecular subtypes (CMS) compared to the normal colon, with significantly higher overexpression in CMS2 [15], which is characterized by high Wnt and Myc signaling activation [23]. However, no information is available on the molecular mechanisms involved in *THRA* regulation in the context of CRC.

Interestingly, the *THRA* gene was characterized in the early 1990s as the cellular homolog of the avian retroviral erytroblastoma virus *v‐erbA*, which is involved in neoplastic transformation leading to acute erythroleukemia and sarcomas [24, 25], thus suggesting its link with malignancies. However, very few studies have analyzed the genomic organization and transcriptional control of the *THRA* gene [26, 27]. Ishida et al. [28] observed that the 615‐bp 5′‐flanking sequence of the *THRA* promoter presented putative binding sites for several transcription factors, including SP1, cAMP‐responsive elements (CRE), CREB, AP1, Krow‐20, COUP‐TF/EAR‐3, and retinoid X receptor (RXR) [28]. Another study described the regulation of the *THRA* gene promoter by the orphan nuclear receptor ERRα [29]. However, these reports did not consider cell type‐specific control under physiological or pathological conditions.

In the current study, we investigated the mechanisms underlying *THRA* transcriptional regulation, including the modulation of the TRα1 receptor. *In silico* and molecular approaches identified promoter regions and transcription factors important for *THRA* activity. We demonstrated the presence of binding sites for transcription factors involved in intestinal homeostasis and SC/cancer SC biology that are also altered in CRC [30, 31, 32], such as TCF7L2 (Wnt pathway) [33], RBPJ (Notch pathway) [34], and CDX2 (intestinal epithelial cell identity) [35]. Finally, in‐depth analysis of the Wnt pathway allowed us to recapitulate the regulation of *THRA* transcription and TRα1 expression by this signaling pathway in human adenocarcinoma cell lines as well as mouse enteroids. This study presents the first extended analysis of *THRA* regulation and its relevance in a patho‐physiological context. In addition, it describes, for the first time, the existence of a reciprocal regulatory loop between TRα1‐dependent and Wnt‐dependent signals in intestinal epithelial cells.

## 2 Materials and methods

### 2.1 Tissue microarray analysis (TMA)

TRα1 expression has been analyzed by immunohistochemistry on Tissue Focus Colon Cancer Tissue MicroArray, FFPE, 42 × 1 mm cores (CT565864; CliniScences, Nanterres, France). The TMA was composed of 33 tumors at different stages and 9 normal tissues. The study and label scoring were conducted by the Research Pathology Platform (Lyon, France). Briefly, after deparaffinization and dehydration, tissue sections were heated for 50 min at 97 °C in 10 mm citrate buffer, pH 6.0. To block endogenous peroxidases, tissue sections were incubated in 5% hydrogen peroxide solution. Immunohistochemistry (IHC) was performed on an automated immunostainer (Ventana Discovery XT; Roche, Meylan, France) using an Omnimap DAB Kit (Ventana Medical Systems, Tucson, AZ, USA) according to the manufacturer's instructions. Sections were incubated with the anti‐TRα1 antibody (ab53729, dilution 1 : 50). The secondary anti‐rabbit‐HRP antibody was applied to the sections, and staining was visualized with DAB solution with 3,3′‐diaminobenzidine as a chromogenic substrate. Finally, the sections were counterstained with Gill's hematoxylin and then scanned with a Panoramic Scan II (3D Histech, Budapest, Hungary) at 20×. The scoring of TRα1 levels (−, negative; +/−, low; + positive; ++, highly positive) was performed independently by two individuals.

### 2.2 Bioinformatics analyses of the TGCA CRC cohort

To analyze the expression levels of THRA in the TCGA cohort, RNA sequencing data from 270 colon adenocarcinoma (COAD) and 41 adjacent normal samples were obtained from the TCGA data portal (https://portal.gdc.cancer.gov/). To obtain Transcripts Per Million (TPM) normalized expression levels of the *THRA* canonical transcript, we used Kallisto [36] with GENCODE (https://www.gencodegenes.org; v29) as reference to the human transcriptome. TCGA samples were also classified according to CMSs [23] using the R package CMS classifier (v1.0.0). Boxplots were created using the R packages ggplot2 (v3.3.2) and ggpubr (v0.4.0), and comparisons between groups were assessed by Wilcoxon tests.

### 2.3 *In silico* *THRA* promoter analysis

Analysis of approximately 3500 bp of the *THRA* promoter region upstream the transcription start site (TSS) was performed by the MatInspector library (Genomatix, Munich, Germany), using Matrix Family Library Version 11.0. Filters were applied to select a core matrix similarity > 0.85 (85% of conserved homology) using the module General Core Promoter Elements (Optimized).

### 2.4 Construction of the *THRA* ‐luciferase vectors

3238‐bp upstream of the transcription starting site of the *THRA* gene were cloned into the pGL3 basic vector (Promega, Charbonniere‐les‐Bains, France) to construct the pGL3‐*THRA*‐Luc vector (named pGL3‐*THRA*) using MluI (5′) and XhoI (3′) sites (Fig. S1A). A CT>GC mutation was introduced at positions 816 and 2270 of the pGL3‐*THRA*‐luc vector, separately, to mutate the TCF7L2‐binding sites and generate the *THRA*‐mut‐Luc1 and *THRA*‐mut‐Luc2 vectors (named pGL3‐*THRA*‐mut‐TCF7L2‐1 and pGL3‐*THRA*‐mut‐TCF7L2‐2, respectively) (Fig. S1B,C). For the generation of the double mutant vector (named pGL3‐*THRA*‐mut‐TCF7L2‐sites, Fig. S1D), both mutant plasmids were digested with AvrII and StuI enzymes (New England Biolabs, Evry, France). The fragment containing the mutant TCF7L2‐1 site was ligated into the vector containing the mutant TCF7L2‐2 site using DNA quick ligase (M2200L; New England Biolabs). The ligated mix was used to transform competent bacteria, and the colonies were recovered for DNA plasmid preparation and sequencing. Gene synthesis, site‐directed mutagenesis, and sequencing were performed by Eurofins Genomics (Ebersberg, Germany).

### 2.5 Cell lines and transfection experiments

The human adenocarcinoma cell lines Caco2, HCT116, and SW480 (from ATCC, Rockville, USA) were cultured in DMEM Glutamax (4.5 g·L^−1^ d‐Glucose with pyruvate) medium (ThermoFisher Scientific, Courtaboeuf, France) supplemented with 10% heat‐inactivated FBS and 1% penicillin/streptomycin (P/S) (ThermoFisher Scientific) at 37 °C in a humidified atmosphere containing 5% CO_2_.

For luciferase assays, we seeded each cell line onto 24‐well plates (75 000 cells/well) in DMEM supplemented with 10% FBS and 1% P/S. The next day, we transfected the plasmids using PEI Prime™ linear polyethylenimine (Sigma‐Aldrich 919012; Saint‐Quentin Fallavier, France) at a ratio of 1 μg DNA/1.5 μL of PEI at 1 mg·mL^−1^. Transfection was performed for 6 h in culture medium without serum. Luciferase activity was measured 48 h after transfection using the Dual‐Luciferase Reporter Assay System (Promega). Data represent the normalized beetle‐luciferase/renilla‐luciferase activities measured in each well to correct for eventual differences in transfection efficiency from well to well. Experiments were performed at least two times with *n* = 6 for each condition.

#### 2.5.1 Luciferase reporter vectors

*THRA*‐Luc (200 ng/well), *THRA‐mut1‐Luc, THRA‐mut2‐luc and THRA‐dmut‐luc* (200 ng/well), TopFlash (200 ng/well; Fisher Scientific, Illkirch, France), *RBPJ*‐Luc (200 ng/well, [37]), *hLI*‐Luc (200 ng/well), pGL3‐basic (200 ng/well), and pRL‐CMV (10 ng/well; Promega) were used. The generation of *hLI*‐Luc was based on a previous publication [38] and consisted of cloning approximately 1 kb of the human LI‐cadherin gene promoter into the pGL3 basic vector using Sac1 (5′) and HindIII (3′) restriction sites.

#### 2.5.2 Expression vectors

β‐Catenin ΔN (100 ng per well, gift from Pr M. Waterman), TCF1E‐EVR2 (100 ng per well) [39], TCF1E‐EVR2‐DN (300 ng per well) [39], CDX2 (100 ng per well) [40], and NICD (100 ng per well) [37] were used. The amounts of DNA under each condition were normalized by adding the empty pBSK vector. The experiments based on Wnt blocking and restimulation were performed by transfecting the TCF1‐DN vector (dominant negative; 300 ng per well) in the absence or presence of increasing amounts of β‐catenin ΔN expression vector (from 50 to 500 ng per well).

#### 2.5.3 siRNA approach

For CDX2 expression modulation by the siRNA approach, we seeded each cell line into 24‐well plates (75 000 cells per well) in DMEM supplemented with 10% FBS and 1% P/S. The next day, we removed the medium and added siRNA CDX2 (Silencer^®^ Select siRNA@CDX2 s2876; ThermoFischer) or the siRNA control (Silencer™ Select Negative Control No. 2 siRNA; ThermoFischer, #4390846) at a final concentration of 10 nm in a mix containing OPTIMEM medium (ThermoFisher Scientific) and lipofectamine RNAiMAX (ThermoFisher Scientific) for 24 h. In CDX2‐KD and control cells, we performed *THRA*‐luc transfection assays as indicated above.

#### 2.5.4 Treatments with small molecules

For modulation of the Wnt pathway, we used an approach consisting of treatment with small molecules. Transfected cells, as described above were treated with each molecule 24 h before the end of transfection. We used the Wnt agonists CHIR99021, 3 μm (Sigma‐Aldrich) [41] and the Wnt antagonist IWP4, 5 μm (Tocris, Noyal Chatillon sur Seiche, France) [42]; the Notch agonist Yhhu3792, 2.5 μm (Tocris) [43]; and the Notch antagonists LY411575, 1 μm (Sigma‐Aldrich, Saint‐Quentin‐Fallavier, France) [44], and DAPT, 10 μm (Tocris) [45]. The effect of the Wnt agonist and antagonist on endogenous TRα1 expression was analyzed by treating the cells for 48 h before harvesting.

### 2.6 ChIP and qPCR analysis

For ChIP experiments, each cell line was seeded in 6 cm dishes, and the cells recovered after 2 days of culture. Chromatin crosslinking was performed with 1% (vol/vol) formaldehyde for 10 min at room temperature and quenched with 0.125 mol·L^−1^ glycine for 5 min. ChIP experiments were performed using the EZ‐Magna ChIP G Chromatin Immunoprecipitation kit (Sigma‐Aldrich, #17‐409) as recommended by the supplier. Sonicated chromatin (BioruptorPlus, Diagenod apparatus, Seraing, Belgium; 12 cycles of 30 s ON/30 s OFF on high mode) from 2 × 10^6^ cells was incubated overnight at 4 °C with 4 μg of mouse anti‐β‐catenin antibody (clone 14; BD Transduction Lab, Le Pont de Claix, France) or with immunoglobulin G (IgG) (Cell Signaling, Leiden, The Netherlands). DNA was quantitated by qPCR using SYBR qPCR Premix Ex Taq II (Tli RNaseH Plus; Takara, Saint‐Germain‐en‐Laye, France) in a CFX Connect apparatus (Bio‐Rad, Marnes‐la‐Coquette, France). The primers used for AXIN2, MYC, THRA‐1, THRA‐2, PPIB, and HPRT are listed in Table S1. The antibodies are listed in Table S2. Histograms represent the fold enrichment of specific β‐catenin DNA binding normalized to the input and compared with the IgG condition (= 1).

### 2.7 Western blot

Protein samples from each cell line (30 μg per lane) were prepared with RIPA buffer as described in [15], separated by SDS/PAGE, and transferred to 0.2‐μm PVDF membranes (Bio‐Rad). Membranes were blocked with PBS‐Tween supplemented with 5% nonfat milk before incubation with primary antibodies. This step was followed by incubation with HRP‐conjugated secondary antibodies (Promega). The signal was analyzed using an enzymatic Clarity Substrate Detection Kit and Clarity Max ECL (Bio‐Rad) according to the manufacturer's protocol, and image detection was performed using a Pixie imaging system (Gene‐sys, France). The antibodies are listed in Table S2.

### 2.8 Animals, isolation of small intestinal crypts, and enteroid cultures

Villin‐Cre^ERT2^ and Apc^+/fl^ mice have been bred in our laboratory since 2009, when they were provided by the Institut Curie (Paris, France) [46, 47]. For our study, adult 2–4‐month‐old Apc^+/fl^/Villin‐Cre^ERT2^ and Apc^+/fl^ mice were maintained in a C57BL/6J genetic background and housed in the same animal facility, where they received standard mouse chow and water *ad libitum*. All experiments were performed in compliance with the French and European guidelines for experimental animal studies and approved by the local committees “Comités d'Éthique Ceccapp” (C2EA55) “the Ministère de l'Enseignement Supérieur et de la Recherche, Direction Générale pour la Recherche et l'Innovation, Secrétariat “Autorisation de projet” (agreement # 13313‐2017020210367606).

After sacrifice, we collected the small intestine (from the proximal jejunum to the distal ileum) for crypt preparation and enteroid cultures, using the protocol previously described [13]. Organoids were cultured at 37 °C and 5% CO_2_ in IntestiCult Organoid Growth Medium (Stem Cell Technologies, Saint Egreve, France). The medium was changed every 3 days, and organoids were replicated approximately 7 days after the beginning of the culture. For all experiments (three independent experiments from three independent mice), we used organoids after the first replication (R1). Briefly, Matrigel‐embedded organoids were grossly dissociated with a micropipette, fragments were washed in PBS and recovered by centrifugation. They were mixed with Intesticult/Matrigel mix (1 : 1 volume), plated in 50 μL drops, and covered with 900 μL of culture medium in 12‐well plates. Twenty‐four hours after replication, organoids were treated with 4‐OH‐tamoxifen (0.2 mg·mL^−1^; Sigma‐Aldrich H6278) or DMSO (control) for 24 h and monitored for several days after treatment. The cultures were recovered on day 5 for genomic DNA (gDNA) and RNA extraction. Pictures were taken over the days of culture using a Zeiss AxioVert (Marly le roi, France) inverted microscope with a 10× objective.

### 2.9 Genomic DNA extraction and PCR analysis

We extracted gDNA from Apc^+/fl/^Villin‐Cre^ERT2^ and Apc^+/fl^ enteroids at D5 using the Nucleospin Genomic DNA from Tissue kit (Machery‐Nagel). The presence of the *Apc*‐mutated allele was detected by PCR using specific primers listed in Table S1.

### 2.10 RNA extraction and RTqPCR

We extracted total RNA using the Nucleospin RNA Kit (Macherey‐Nagel, Hoerdt, France). We performed DNase digestion on all samples to remove contaminating gDNA and reverse transcription (RT) of total RNA with iScript reverse transcriptase (Bio‐Rad), according to the manufacturer's instructions. We conducted PCR on all preparations to amplify a housekeeping gene (*Hprt/HPRT*) for which the primers are located on different exons of the corresponding gene to further exclude gDNA contamination after RT. For qPCR approaches, we used SYBR qPCR Premix Ex Taq II (Tli RNaseH Plus; Takara) in a CFX Connect apparatus (Bio‐Rad). In each sample, specific mRNA expression was quantitated using the ΔΔ*C*_t_ method, and values were normalized against *Ppib/PPIB* levels. The primers used are listed in Table S1.

### 2.11 Statistical analysis

Statistical analyses were conducted using graphpad prism software (version 8; GraphPad Software Inc., San Diego, CA, USA), and the level of significance was established as *P*‐value < 0.05.

## 3 Results

### 3.1 Expression of *THRA* in human colorectal cancer

**Fig. 1:**
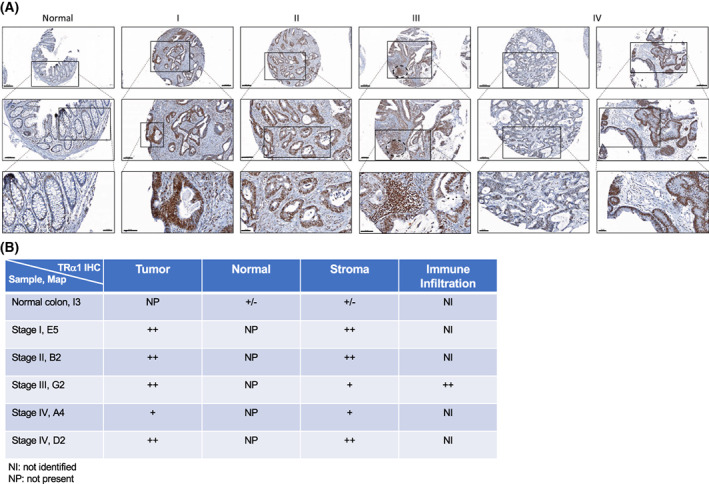
Expression of TRα1 in human TMA of colorectal samples. (A) Immunohistochemical analysis of TRα1 expression in a CRC cohort of patients with different indicated tumor stages (I, II, III, and IV) and in the normal colon. Scale bar: 200 μm (low magnification), 100 μm (medium magnification), 50 μm (high magnification). (B) Scoring of TRα1 protein levels: −, negative; +/−, low; +, positive; ++, highly positive. The scoring was performed independently by two individuals.

Our previous studies showed increased expression of the TRα1 protein in CRC from patients compared with the normal colon [15]. Here, we enlarged this study and used an approach of IHC on a tissue microarray (TMA) to analyze TRα1 expression in a CRC cohort of patients with different tumor stages (Fig. 1, Fig. S2; Table S3 summarizes all known characteristics of the samples). TRα1 labeling intensity was scored in each sample, including the tumor and normal tissue as well as the stromal cells and the immune infiltrate (Fig. 1B, Table S3). Compared with the normal colon, TRα1 immunolabeling was clearly stronger in almost all tumors and at all tumor stages. Even if a clear difference was not observed based on the tumor stage, we noticed that stage II and stage III tumor samples frequently presented TRα1‐positive immune infiltrating cells (Fig. 1, Table S3), as determined by morphological characteristics [48, 49]. It is worth emphasizing, however, that evident intratumor heterogeneity was observed, with some cells or tumoral parts strongly labeled and some cells or tumoral zones more lightly labeled or negative. In addition, high‐magnification images enabled us to distinguish stromal cells expressing different levels of TRα1 and TRα1‐negative cells. In stage IV CRC, we could identify tumors displaying variable levels of TRα1 (i.e., in comparing the two images of stage IV), clearly indicating intertumor heterogeneity of TRα1 expression. *THRA* gene expression was also determined in the human TCGA COAD cohort (Fig. S3). Despite the absence of a difference when globally comparing CRCs and normal tissues likely due to the high heterogeneity (Fig. S3A), we confirmed increased *THRA* expression in the CMS2 high‐Wnt molecular subtype [23] (Fig. S3B), as previously described in another cohort [15].

Altogether, these results reinforce our previous data showing that TRα1 is upregulated in human CRC and is correlated with Wnt pathway activity.

### 3.2 *THRA* promoter analysis

**Fig. 2:**
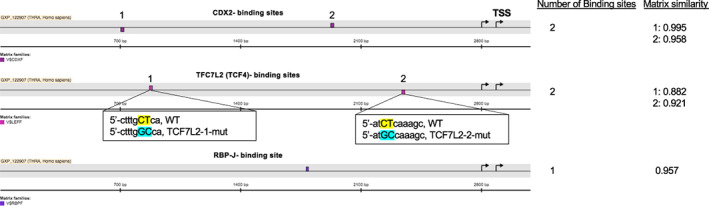
*THRA* promoter *in silico* analysis. *In silico* analysis of 3238 bp of the *THRA* promoter revealed the presence of binding sites for different transcription factors, such as CDX2, TCF7L2, and RBPJ. The insets below the TCF7L2‐binding sites show the changes introduced in the mutant promoters compared to the WT. The approximate location of the binding sites in the scheme is assigned from the 5′ portion of the promoter. On the right, the number of putative binding sites and the matrix similarity for each site compared with the canonical binding site are indicated (1 = 100% similarity).

Because of the upregulation of *THRA* in the CRC cohorts and the specific expression of TRα1 in intestinal crypt cells [14], we wanted to determine the molecular basis of its expression regulation. *In silico* analysis of 3238 bp of the *THRA* promoter region showed the presence of several putative binding sites for transcription factors, such as TCF7L2, RBPJ, and CDX2, that are fundamental to intestinal physiology and are altered in CRC [50] (Fig. 2). Of note, other studies have also shown β‐catenin‐binding sites in the boundaries of the *THRA* gene [51]. The *THRA* promoter region was cloned into a luciferase reporter vector (*THRA*‐luc, Fig. S1A), and its activity was analyzed in transient transfection experiments. We implemented two steps to take into account the genetic heterogeneity of CRC and avoid bias in the experiments. First, we performed the study using three different human COAD cell lines—Caco2, SW480, and HCT116—displaying different mutations of genes or pathways that are more frequently altered in CRC [52, 53, 54, 55] (Fig. S4A). Second, all cell lines were maintained in culture medium supplemented with the same concentration of serum, ensuring comparable amounts of growth factors that could potentially influence subsequent analyses [56]. We also verified TRα1 expression by RTqPCR and noticed that SW480 cells presented significantly higher mRNA levels than Caco2 and HCT116 cells.

**Fig. 3:**
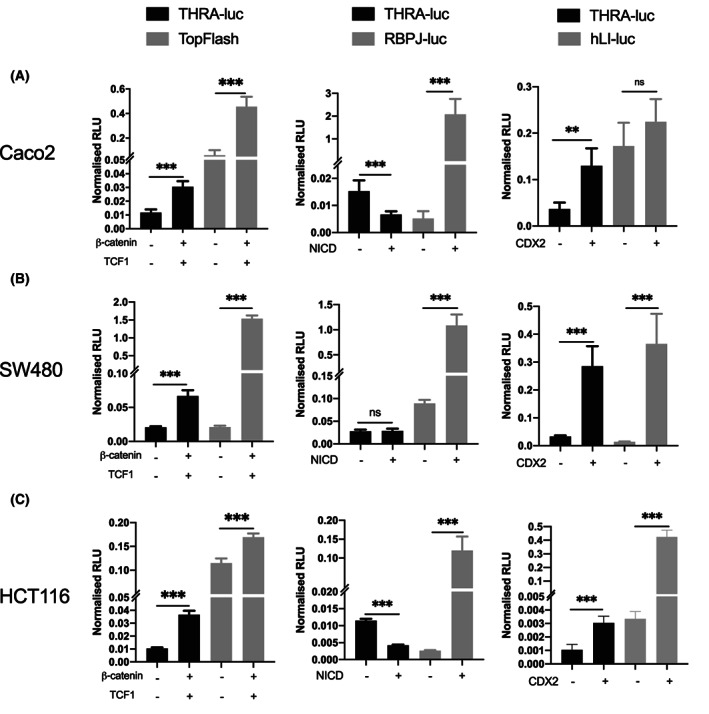
Modulation of *THRA* promoter activity in human adenocarcinoma cell lines by Wnt, notch, and CDX2. (A–C) Caco2 (A), SW480 (B), and HCT116 (C) cells were transfected with the *THRA*‐luc vector alone or cotransfected with different transcription factors. The left panels show results obtained with the Wnt cofactors β‐catenin/TCF1; the central panels show results obtained with the notch pathway activator NICD; and the right panels show results obtained with CDX2. TopFlash, *RBPJ*‐luc, and *hLI*‐luc were used as positive controls for Wnt, notch, and CDX2, respectively. Graphs show the mean ± SD (*n* = 6) of normalized relative luciferase units (RLU) from at least two independent experiments, each conducted in six replicates. ns, nonsignificant, **P < 0.01, and ***P < 0.001 by unpaired, two‐tailed Student's *t*‐test.

When we started the promoter analysis, we observed *THRA*‐dependent luciferase basal activity in every cell line, compared with the pGL3‐basic vector (Fig. S5A). In addition, upon cotransfection with the Wnt transcriptional regulators β‐catenin/TCF1, regardless of the mutational background of the cells, the activity of the *THRA* promoter was significantly increased (Fig. 3A–C, left panels). The results on the Notch pathway relative to the *THRA* promoter were more complex, as cotransfection of the Notch intracellular domain (NICD) decreased the luciferase activity in Caco2 and HCT116 cells but had no effect on SW480 cells (Fig. 3A–C, middle panels). When we analyzed the effect of CDX2 on *THRA*, we observed positive regulation of luciferase activity in all cell lines (Fig. 3A–C, right panels). The TopFlash, *RBPJ*‐luc, and *hLI*‐luc vectors were used, respectively, as the positive controls for Wnt, Notch, and CDX2 activities. Finally, no effect of the transcription factors could be detected when using the pGL3‐basic vector (Fig. S5B).

Taken together, these data show that *THRA* promoter activity is positively regulated by the Wnt/β‐catenin pathway and CDX2 in human colorectal adenocarcinoma cell lines. The Notch pathway plays a more complex role and behaves as a negative regulator or has no effect on *THRA* promoter.

### 3.3 Analysis of *THRA* promoter upon modulation of CDX2 expression and signaling pathway activity

**Fig. 4:**
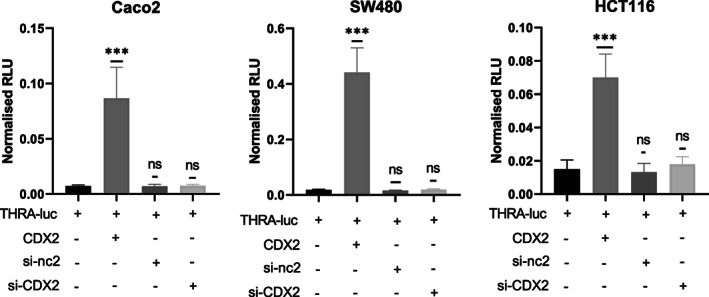
Effect of CDX2‐KD on *THRA*‐luc activity. Analysis of the effect of silencing CDX2 on the *THRA* promoter activity in the adenocarcinoma cell lines Caco2, SW480, and HCT116, as indicated. Graphs show the mean ± SD (*n* = 6) of normalized relative luciferase units (RLU) from at least two independent experiments, each conducted in six replicates. ns, nonsignificant and ***P < 0.001 by unpaired, two‐tailed Student's *t*‐test.

To further link *THRA* promoter activity with Wnt, Notch, and CDX2, we used approaches involving modulation by siRNA (CDX2) or small molecules (Wnt and Notch). In the case of CDX2, we confirmed its stimulatory effect on *THRA* activity, which was lost in CDX2‐KD cells transfected with siRNA@CDX2 (Fig. 4). However, siRNA@CDX2 *per se* did not decrease *THRA* activity (Fig. 4). Treatment of the cell lines with Notch agonists or antagonists confirmed the complex scenario observed in the cotransfection experiments described in the previous paragraph (not shown). We then decided to focus specifically on more in‐depth analysis of the Wnt pathway, considering the cross‐talk and synergy between TRα1 and Wnt reported in previous studies [14, 15, 57].

**Fig. 5:**
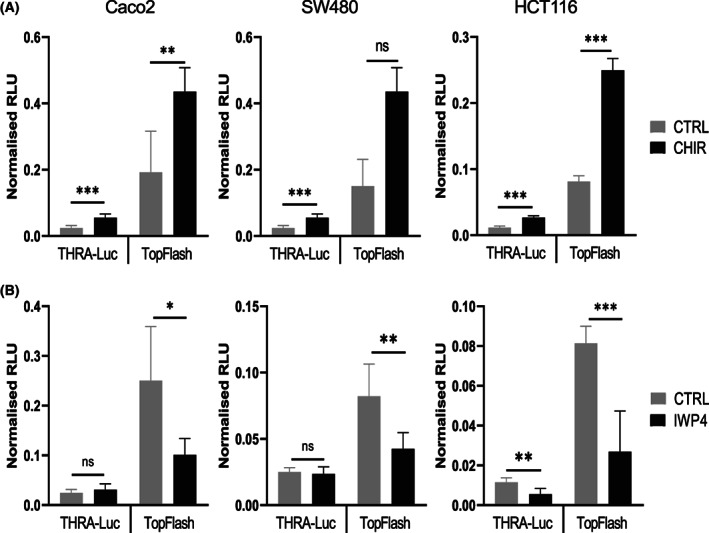
Modulation of *THRA*‐luc activity by the Wnt agonist and antagonist. Analysis of *THRA*‐luc and TopFlash activity in the presence of the Wnt agonist CHIR99021 (A) and the Wnt antagonist IWP4 (B) in Caco2, SW480, and HCT116 cells, as indicated. Graphs show the mean ± SD (*n* = 6) of normalized relative luciferase units (RLU) from at least two independent experiments, each conducted in six replicates. ns, nonsignificant, *P < 0.05, **P < 0.01, and ***P < 0.001 by unpaired, two‐tailed Student's *t*‐test.

The three cell lines were treated with the Wnt activator CHIR99021 (CHIR) [41] or the Wnt antagonist IWP4 for 24 h [42]. As expected, CHIR increased the activity of both *THRA*‐luc and TopFlash (Fig. 5A). IWP4 induced a significant decrease in TopFlash activity in all cell lines compared with the control (Fig. 5B). However, when we analyzed the action of this molecule, it clearly inhibited *THRA*‐luc activity only in HCT116 cells (Fig. 5B). We also evaluated the effect of the Wnt modulators on endogenous TRα1 expression and compared it with the cotransfection of β‐catenin and TCF1 (Figs S6 and S7). Although we confirmed a difference in the response to Wnt modulators depending on the cell line (Fig. S6), the cotransfection of cells with β‐catenin/TCF1 resulted in increased TRα1 mRNA and protein expression in all cell lines (Fig. S7A,B).

Overall, we confirmed that high CDX2 levels regulated the *THRA* promoter and that its activity was increased by the Wnt agonist CHIR. In addition, Wnt stimulation also affected endogenous TRα1 expression.

### 3.4 Mutational and functional analyses of the *THRA* promoter

**Fig. 6:**
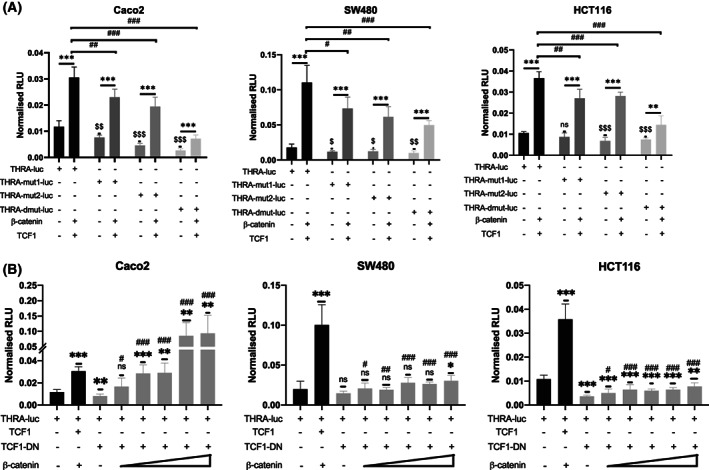
Mutations and Wnt‐blocking analyses on *THRA* promoter activity. (A) TCF7L2‐binding site mutations. *THRA*‐luc or the different *THRA*‐Mut‐luc constructs, as indicated, were transfected alone or cotransfected with β‐catenin and TCF1. Experiments were performed in Caco2 (left panel), SW480 (central panel), and HCT116 (right panel) cells. The graphs show the mean ± SD (*n* = 6) of the normalized relative luciferase units (RLUs) from at least two independent experiments, each of which were conducted with six replicates. ns, nonsignificant, **P < 0.01, and ***P < 0.001 comparing the basal activity with the activity after cotransfection. ^#^ *P* < 0.05, ^##^ *P* < 0.01 and ^###^ *P* < 0.001 comparing *THRA*‐luc with the different mutated constructs after cotransfection with β‐catenin/TCF1. ^$^ *P* < 0.05, ^$$^ *P* < 0.01 and ^$$$^ *P* < 0.001 comparing the basal activity of *THRA*‐luc with that of the different mutated constructs. Statistics were performed using unpaired, two‐tailed Student's *t*‐test. (B) The *THRA*‐luc construct was transfected alone or cotransfected with TCF1‐DN and different amounts of β‐catenin (0, 50, 100, 200, 300, and 500 ng), as indicated. Experiments were performed in Caco2 (left panel), SW480 (central panel), and HCT116 (right panel) cells. The graphs show the mean ± SD (*n* = 6) of the normalized relative luciferase units (RLUs) from at least two independent experiments, each of which were conducted with six replicates. ns, nonsignificant, *P < 0.05, **P < 0.01, and ***P < 0.001 compared with the basal promoter activity. ns, nonsignificant, ^#^ *P* < 0.05, ^##^ *P* < 0.01, and ^###^ *P* < 0.001 by comparing the activity of *THRA*‐luc cotransfected with TCF1‐DN alone with the activity in the presence of different concentrations of β‐catenin. Statistical analysis was performed based on an unpaired, two‐tailed Student's *t*‐test.

To definitively link the *THRA* promoter activity with the Wnt/β‐catenin signaling pathway, we performed experiments on the *THRA*‐luc construct carrying a mutation in each of the TCF7L2 sites (*THRA*‐mut1‐luc and *THRA*‐mut2‐luc vectors) or in both TCF7L2 sites (*THRA*‐dmut‐luc vector) (Fig. S1B–D). We performed experiments in parallel with *THRA*‐luc and the mutated versions in the three cell lines used in the previous experimental protocols in the presence or absence of cotransfected β‐catenin/TCF1 (Fig. 6A). In both *THRA*‐mut1‐luc and *THRA*‐mut2‐luc, the induction of *THRA*‐dependent luciferase activity by β‐catenin/TFC1 significantly decreased in the three cell lines compared to that observed with the WT promoter (Fig. 6A). This effect was even more evident when using the double mutant vector, as the induction of *THRA*‐dependent luciferase activity by β‐catenin/TFC1 was strongly affected in all cell lines. Importantly, the mutations in each or both TCF7L2 sites decreased the *THRA*‐dependent luciferase basal activity in all cell lines compared with the nonmutated promoter.

To further confirm the importance of Wnt activity on the *THRA* promoter, we also performed experiments using a vector expressing a mutated form of TCF1 that acts as a dominant‐negative (TCF1‐DN) vis‐à‐vis the WT protein [58]. By cotransfecting the TCF1‐DN vector, we observed a significant decrease in *THRA*‐luc activity in Caco2 and HCT116 cells but only a slight decrease in THRA‐luc activity in SW480 cells (Fig. 6B). When we re‐expressed increasing amounts of β‐catenin (from 50 ng to 500 ng) under suppressed Wnt conditions, we observed an increased *THRA*‐luc activity that differed among the cell lines. In Caco2 cells, a dose–response effect to increased β‐catenin amounts was observed. In SW480 and HCT116 cells, the luciferase activity increased significantly compared with the TCF1‐DN condition but rapidly reached a plateau and could not be stimulated by higher β‐catenin concentrations (Fig. 6B). The efficacy of TCF1‐DN was validated using the TopFlash control vector (Fig. S8).

**Fig. 7:**
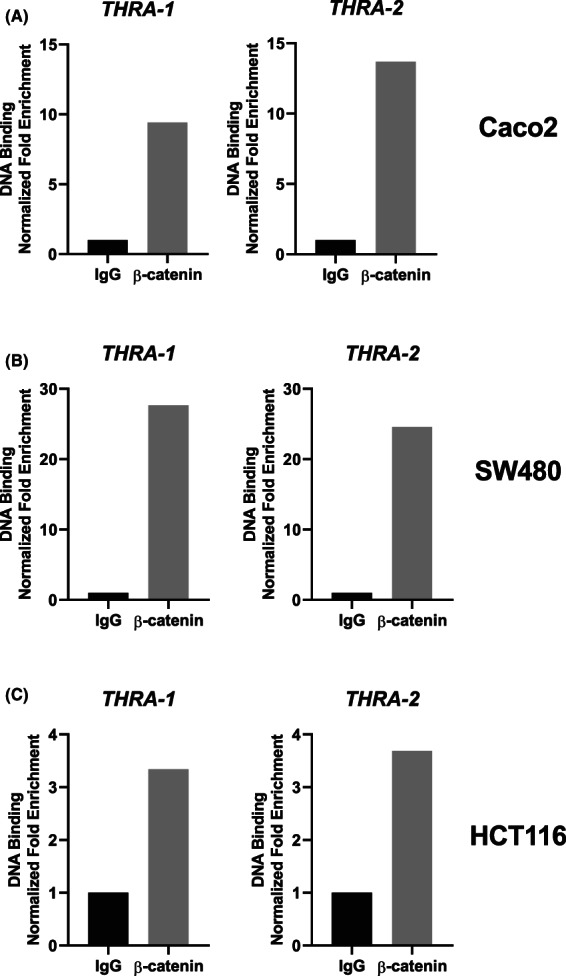
Chromatin occupancy of β‐catenin in the *THRA* gene promoter. ChIP analysis was performed with chromatin prepared from (A) Caco2, (B) SW480, and (C) HCT116 cells and immunoprecipitated using an anti‐β‐catenin or IgG (negative control). qPCR was performed using specific primers covering each TCF7L2‐binding site within 3 kb of the *THRA* promoter. The results are representative of two independent experiments. Histograms represent the fold enrichment of specific β‐catenin/DNA binding normalized to the input and compared with the IgG condition (= 1).

The above results and those described in the previous paragraphs prompted us to determine whether the regulation of the *THRA* promoter by the Wnt effectors β‐catenin/TCF was mediated by direct binding to chromatin. For this aim, we used a ChIP approach in the three cell lines. ChIP was performed by using anti‐β‐catenin or IgG (negative control). As shown in Fig. 7, β‐catenin bound to the *THRA* promoter regions containing the TCF7L2 sites. The specificity of β‐catenin binding was validated on *AXIN2‐* and *MYC*‐positive control promoters (Fig. S9), whereas no specific binding was detected on the *HPRT* or *PPIB* genes (Fig. S9).

These results underline the control of the *THRA* promoter by the Wnt/β‐catenin pathway in human COAD cell lines, which is exerted through the functional TCF7L2‐binding sites located 3 kb upstream of the transcription start site. In addition, promoter control is achieved by direct binding of β‐catenin to chromatin.

### 3.5 Stimulation of TRα1 expression by activated Wnt in mouse enteroids

The previous results compelled us to investigate the effect of activating Wnt on TRα1 expression in a more complex and physiological model, which eventually recapitulated the steps of Wnt activation in early intestinal lesions. For this purpose, we used Apc^+/fl^/Villin‐Cre^ERT2^ and Apc^+/fl^ mice to generate organoids from the small intestine. In Apc^+/fl^/Villin‐Cre^ERT2^ enteroids, mutation of the *Apc* gene was induced by the addition of 4‐OH‐tamoxifen to the culture medium, resulting in the increase in Wnt activity [46]. Apc^+/fl^ enteroids have been used as negative controls for tamoxifen treatment, given that they do not express the Cre^ERT2^ protein.

**Fig. 8:**
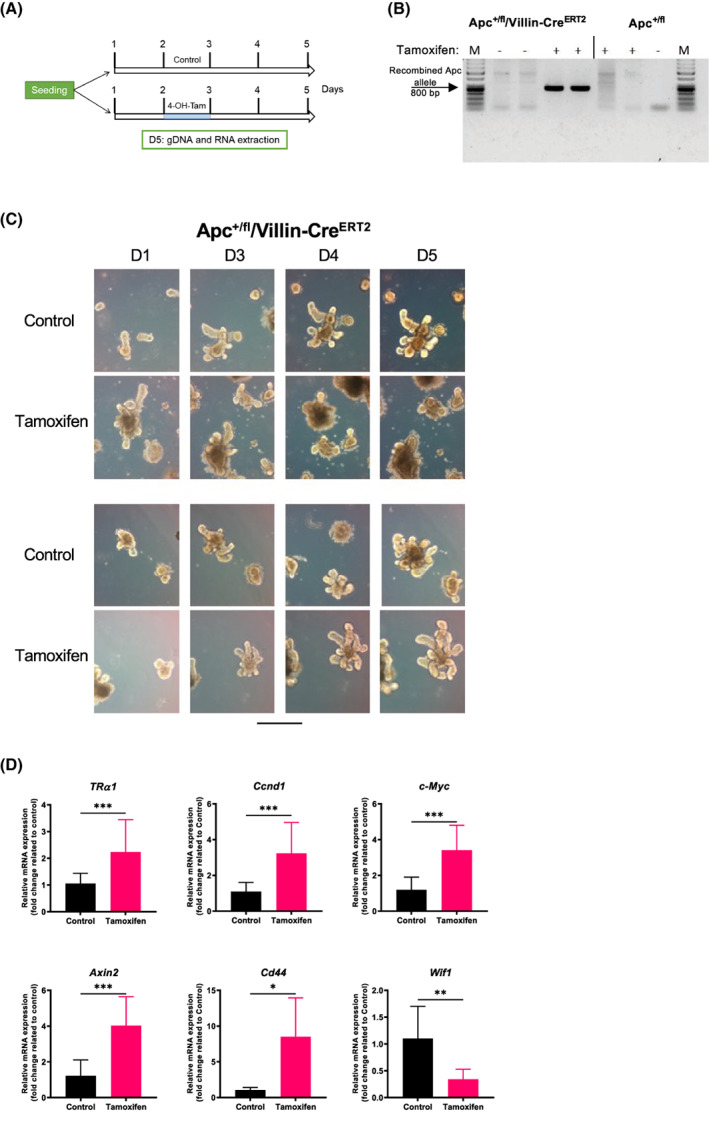
TRα1 modulation by the induction of the Apc mutation in mouse enteroids. (A) Schematic diagram of the protocol used for *ex vivo* enteroid cultures. (B) PCR analysis on gDNA extracted from enteroids of different genotypes and treatments, as indicated, to verify the recombination of the *Apc* gene after tamoxifen treatment. Specific primers recognizing the mutated allele were used. Note that the band corresponding to mutated *Apc* was detected only in Apc^+/fl^/villin‐Cre^ERT2^ tamoxifen‐treated organoids. (C) Bright‐field pictures of enteroids obtained from Apc^f/+^/villin‐Cre^ERT2^ and Apc^+/fl^ mice treated with tamoxifen or not treated (control). Pictures were taken at different days of culture, as indicated, using a Zeiss AxioVert inverted microscope with a 10× objective. Scale bar = 10 μm. (D) RT‐qPCR analysis of the indicated genes performed on RNA isolated from Apc^+/fl^/villin‐Cre^ERT2^ enteroids treated with tamoxifen or not treated (control), as indicated. Histograms represent the mean ± SD (*n* = 6), and data are expressed as the fold change relative to the control condition (= 1). *Ppib* was used as a reference gene. *P < 0.05, **P < 0.01 and ***P < 0.001 by unpaired, two‐tailed Student's *t*‐test. The results in B–D are representative of three independent experiments, each conducted in six replicates.

Enteroids of different genotypes were freshly prepared and cultured for 7 days before replication. One day after replication, they were treated with tamoxifen or DMSO (control) for 24 h (Fig. 8A). The induction of the mutated *Apc* allele by tamoxifen in Apc^+/fl^/Villin‐Cre^ERT2^ enteroids was validated by PCR on genomic DNA, while no effect of tamoxifen was observed in Apc^+/fl^ enteroids (Fig. 8B). The cultures were monitored under a microscope to follow their growth depending on the genotype and conditions for 4 days after treatment (Fig. 8C). In control condition, independent of the genotype, enteroids underwent typical development during the days in culture, characterized by the outgrowth and lengthening of buds (Fig. 8C). Consistent with previous reports [59, 60] upon tamoxifen treatment of Apc^+/fl^/Villin‐Cre^ERT2^ enteroids, we observed a change in their morphology, with a reduced length of buds and enlargement of the central body because of the lack of the Wnt gradient [61] (Fig. 8C, upper panel). On the contrary, tamoxifen treatment of Apc^+/fl^‐derived enteroids produced no obvious changes in their morphology (Fig. 8C, lower panel). We analyzed in these enteroids the expression of *TRα1* and *Wif1*, a negatively regulated direct TRα1 target gene [15], together with a panel of Wnt‐responsive genes. As expected, upon tamoxifen treatment, Apc^+/fl^/Villin‐Cre^ERT2^ enteroids displayed increased mRNA levels of the Wnt targets *Ccnd1, cMyc, Axin2*, and *Cd44* (Fig. 8D). Importantly, in accordance with the data on the promoter analyses, *TRα1* was significantly stimulated in these mutated‐Apc enteroids, and *Wif1* was downregulated (Fig. 8D). RTqPCR analysis on Apc^+/fl^ enteroids showed no effect of tamoxifen treatment (Fig. S10).

## 4 Discussion

It has been more than 50 years since the *THRA* gene was cloned and characterized as a homolog of the *v‐erbA* gene, which is involved in neoplastic transformations leading to acute erythroleukemia and sarcomas [24, 25], strongly suggesting its link with oncogenesis. Because of this peculiarity, it was quite logical to assume that TRα1, which is produced by this locus, behaves as an oncogene. It has also been speculated that a mutated TRα1 instead of the WT form can have a pro‐tumoral function. Indeed, some data have described mutations in the *THRA* gene in gastric cancers (essentially deletions) [62], and mouse models have assigned oncogenic functions to mutated TRα1 [63, 64]. Recent studies by our laboratory, however, clearly indicated the protumoral function of WT TRα1 when overexpressed in the mouse intestine and colon [14]. Studies in human CRC cohorts also allowed us to establish the relevance of observations from mice to human pathology [15]. In this context, the cross‐regulations between TRα1 and the Wnt/β‐catenin pathway are multiple, and in the case of tumor formation and progression, they depend on mutations in the tumor suppressor gene *Apc*/*APC* [14, 15]. Indeed, the *THRA* gene is frequently overexpressed in CRC molecular subtypes, particularly in CMS2 characterized by high Wnt [15]. We would like to emphasize that our previous study also showed its significant association with CMS3, which is characterized by high metabolic status. Differences among the cohorts, in microarray versus RNA‐seq analyses and among the normal counterparts analyzed may account for the discrepancy. Higher TRα1 expression was, however, definitively clear when considering the IHC analysis in the TMA of CRCs, where we observed a strong increase in TRα1 expression in tumors at all stages compared with the normal colon. The results also point to great heterogeneity in tumor parts and/or stromal cells strongly or poorly expressing TRα1. In addition to being upregulated in CRCs, in the normal intestine, TRα1 shows a distinct expression pattern that follows the gradient of Wnt and Notch activities [6, 14]. However, what determines this specific expression domain was unknown and it was also unknown what are the effectors of its increase in CRCs. Of note, only a few studies have analyzed the molecular basis of *THRA* gene regulation [26, 27, 28, 29], and none were performed in the context of cancer. This is the first study analyzing the mechanisms of *THRA* expression regulation in CRCs.

We performed *in silico* analysis on the 3 kb of *THRA* promoter and showed potential binding sites for transcription factors involved in intestinal homeostasis that impact SC biology and CRC development. CDX2 encodes a protein that is a master regulator of intestinal epithelial cell identity [32, 65], and is involved in SC biology [66, 67]. Both tumor inducer and tumor suppressor roles have been indicated for CDX2 [23, 68, 69, 70, 71] and its downregulation is often associated with CRCs [23, 35, 68, 69, 72, 73]. However, in some cases CDX2 has been reported to be overexpressed in CRCs, and in these cases its overexpression stimulates tumorigenesis, suggesting an oncogenic function [74]. We observed here that high CDX2 levels strongly upregulated the *THRA* promoter in all adenocarcinoma cell lines analyzed despite their different genetics and mutation statuses. *Cdx2*‐KO mice display a decreased expression of the *Thra* gene [69], further strengthening our results on the positive control of *THRA* by CDX2. Interestingly, our previous studies showed that *Thra*‐KO mice presented increased *Cdx2* mRNA expression and that *CDX2* promoter activity was blunted by TRα1 in transfection experiments [75, 76]. Finally, our unpublished observations point to a more complex interplay between *TRα1* and *CDX2* in CRC cohorts, as we observed tumors with opposite expression levels of *TRα1* and *CDX2*, as well as tumors displaying a direct correlation between them (both upregulated or downregulated) (M. Plateroti & J.‐N. Freund, personal communication). Future studies will surely shed light on the molecular and cellular mechanisms responsible for this complex interrelation.

The Notch pathway showed intriguing action on the *THRA* promoter, which appears to be dependent upon the cellular context. We observed that NICD transfection decreased *THRA* activity in Caco2 and HCT116 cell lines but had a stimulatory function in SW480 cells. Additionally, the use of small molecules, suggested to behave as agonists or antagonists of Notch, was hampered by the difficulty of definitively assigning specific roles as activators or inhibitors to these molecules. According to the literature, it appears clear that each of their roles is much larger and goes beyond the control of the Notch pathway [72, 73, 74, 75]. It is also worth noting that the Notch pathway has complex cross‐talk with the Wnt pathway, possibly explaining the puzzling results that we observed [22, 37, 76, 77, 78, 79, 80]. Given the regulation of *THRA* gene expression by Wnt (also discussed in the next paragraph), we assume that the three cell lines analyzed, which present different levels of Wnt activity (Fig. S4), might respond differently to Notch, thus explaining the different effects observed on *THRA* activity.

Our previous work described complex cross‐talk between TRα1 and the Wnt pathway [6, 7, 10], but we did not analyze whether Wnt could affect *THRA*/*Thra* expression. Here, we show that in cell lines, activating Wnt in all cases and by all approaches resulted in increased *THRA* promoter activity. This regulation also applied to endogenous TRα1 expression upon β‐catenin/TCF cotransfection. The direct β‐catenin binding of the promoter regions containing TCF7L2‐binding sites strongly supports direct transcriptional regulation. The effect of the Wnt agonist CHIR and Wnt antagonist IWP4 on *THRA* activity and endogenous TRα1 was more complex to analyze. The drugs were chosen based on the literature [41, 42] and validated using the Wnt‐reporter TopFlash in all cell lines. We hypothesize that the differences in responsiveness or lack of responsiveness may depend on the different genetic and epigenetic backgrounds of the cell lines, and these molecules, as previously noted for the Notch agonists and antagonists, could have various targets [77, 78, 79]. In relation to the genetic background with respect to the Wnt pathway, Caco2 cells have a loss‐of‐function (LOF) mutation of the *APC* gene and a silent mutation in the *CTNNB1* gene (coding β‐catenin). SW480 cells have a LOF *APC* mutation and a WT *CTNNB1* gene. HCT116 cells have a gain‐of‐function *CTNNB1* mutation [54, 81]. In addition, these cell lines have different mutations in additional pathways that can also impact Wnt [54, 55], underscoring the complexity encountered when working with these model systems.

**Fig. 9:**
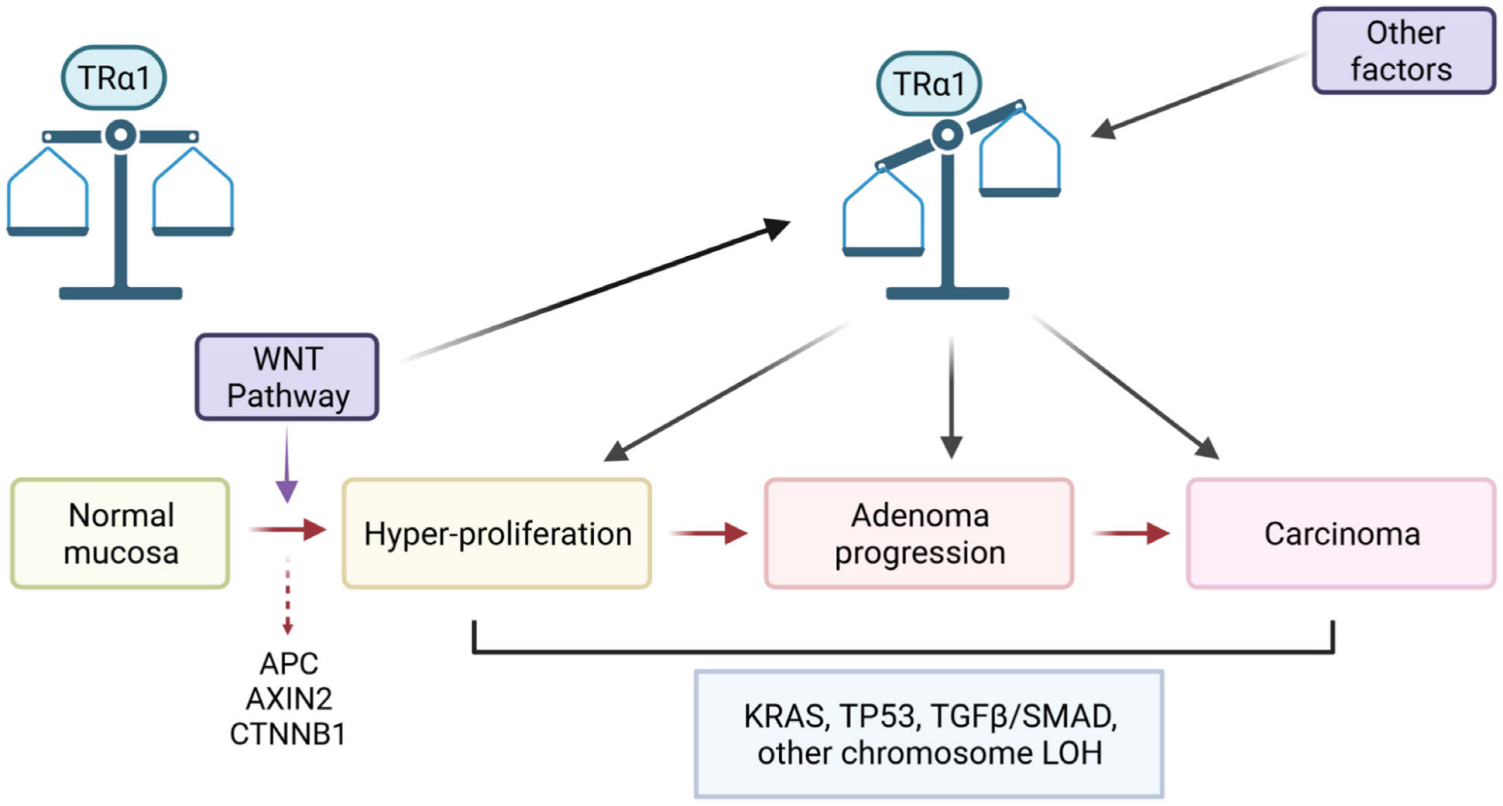
Interplay between TRα1 and the Wnt pathway and correlation with gene deregulation during intestinal tumorigenesis. The picture summarizes the known sequential genetic alterations that are frequently associated with colorectal tumorigenesis in humans. *APC*/*AXIN2*/*CTNNB1* gene mutations, which are responsible for Wnt/β‐catenin overactivation, are key events that occur during the early stage of cell transformation. The other indicated mutations are more frequently associated with later stages [88]. Interestingly, together with the control of the Wnt pathway by TRα1 and its association with the various steps in CRC (hyperproliferation, adenoma progression, and carcinoma generation) [14, 15], our new data point to regulation of the *THRA* promoter by the Wnt pathway and regulation of TRα1 expression by increased Wnt activity in very early stages of tumor development. LOH, loss of heterozygosity. The figure was created with BioRender.com (agreement number: TX23QDYSJV).

Importantly, however, in the physiological context of mouse enteroids, which recapitulate the complexity, organization and hierarchy of the intestinal epithelium [82], stimulating Wnt by *Apc* gene mutation increased TRα1 expression. Altogether, considering our previous and new results, we propose the model illustrated in Fig. 9. High Wnt, as well as other transcription factors, maintains a basal level of TRα1 expression in normal intestinal crypts, where TRα1 integrates and interacts with other key pathways, such as Wnt and Notch, as well as CDX2 [30, 31, 32], to participate in intestinal homeostasis. Upon Wnt overactivation in the early stages of tumor development, TRα1 expression increases, which in turn causes a further increase in Wnt activity responsible for crypt hyperplasia and hyperproliferation, as shown in *vil*‐TRα1 mice [14]. Through its synergy with *Apc*/*APC*‐dependent activated Wnt, TRα1 accelerates tumor growth and participates in tumor progression, including cancer spreading [14] and possibly integrating other tumor processes not yet established. The increased aggressiveness of tumors displaying high TRα1/high Wnt might depend on the strong decrease in the Wnt inhibitors *WIF1*/*Wif1*, *SOX17*/*Sox17*, and *FRZB*/*Frzb* that we have shown in mouse models and patient cohorts [15]. All of these proteins are silenced in CRC, and their silencing characterizes advanced stages and/or more aggressive tumors [83].

## 5 Conclusion

We showed here that several pathways and transcription factors control the expression of the *THRA* gene in the context of CRC. In particular, we unveiled the complex action of the Wnt pathway on *THRA* promoter activity and TRα1 expression. The significance and clinical relevance of high TRα1 expression are of particular interest when considering CRC patients with altered TH levels [84, 85] and/or undergoing chemotherapy treatments that potentially impact thyroid functionality [86, 87].

## Conflict of interest

The authors declare no conflict of interest.

## Author contributions

MVG involved in conception and design, collection and assembly of data, data analyses and interpretation, and manuscript writing; TLR, DF, SB, GDAG, and PAFG involved in collection and assembly of data, data analyses, and interpretation; CD‐D and J‐NF involved in the development of tools, data analyses, and interpretation; MP involved in conception and design, assembly of data, data analyses, and interpretation, manuscript writing, and financial support. All authors approved the manuscript.

## Supporting information


**Fig. S1.** Schematic representation of the *THRA*‐luc constructs.

**Fig. S2.** Setup conditions for TRα1 IHC in human tissue sections.

**Fig. S3.** Analysis of *THRA* expression in a human colorectal cancer cohort.

**Fig. S4.** Characteristics of individual cell lines at multiple molecular levels.

**Fig. S5.** Analyses of the pGL3‐basic vector.

**Fig. S6.** Effect of the Wnt agonist and antagonist on endogenous TRα1 expression.

**Fig. S7.** Effect of β‐catenin/TCF transfection on endogenous TRα1 expression.

**Fig. S8.** TopFlash activity is affected in the presence of TCF1‐DN.

**Fig. S9.** Chromatin occupancy of β‐catenin in the AXIN2 and MYC promoters.

**Fig. S10.** Complementary analysis on mouse enteroids.

**Table S1.** List of primers.

**Table S2.** List of antibodies.

**Table S3.** TMA analysis.

## Data Availability

The data that support the findings of this study are available from the corresponding author (plateroti@unistra.fr) upon request.
