# Supplementary figures and images for: Regulation of the THRA gene, encoding the thyroid hormone nuclear receptor TRα1, in intestinal lesions
Source: Mol Oncol. 2022 Oct 10;16(22):3975–93. doi: 10.1002/1878-0261.13298 (PMC9718118; doi:10.1002/1878-0261.13298)

A

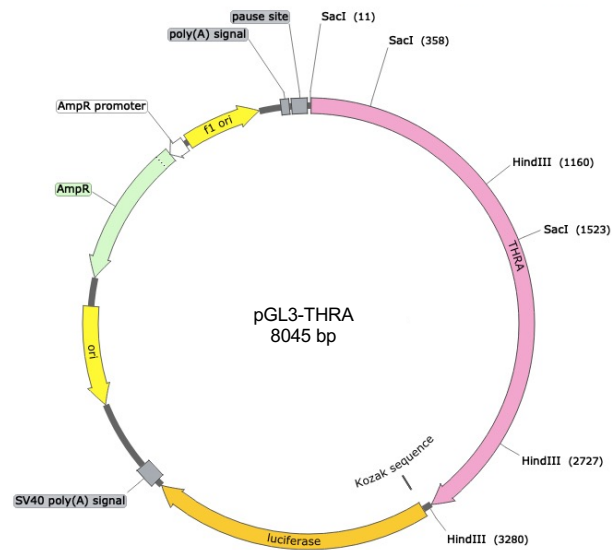

B

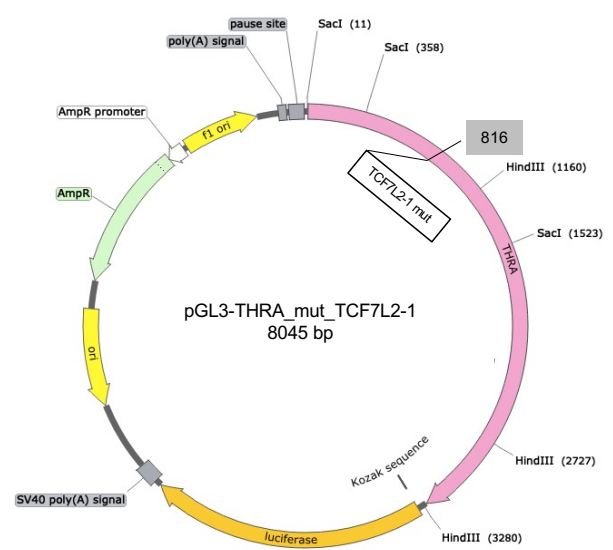

C

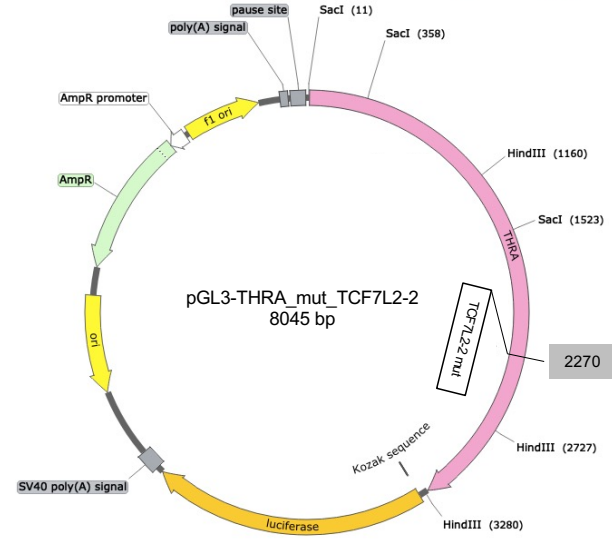

D

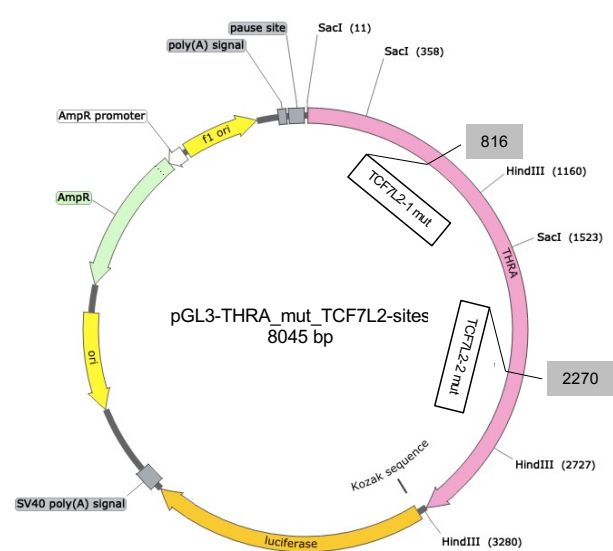

Supplement: Supplementary file 1 — Fig. S1. Schematic representation of the THRA‐luc constructs. [file MOL2-16-3975-s010.pdf]

Giolito et al, Figure S2

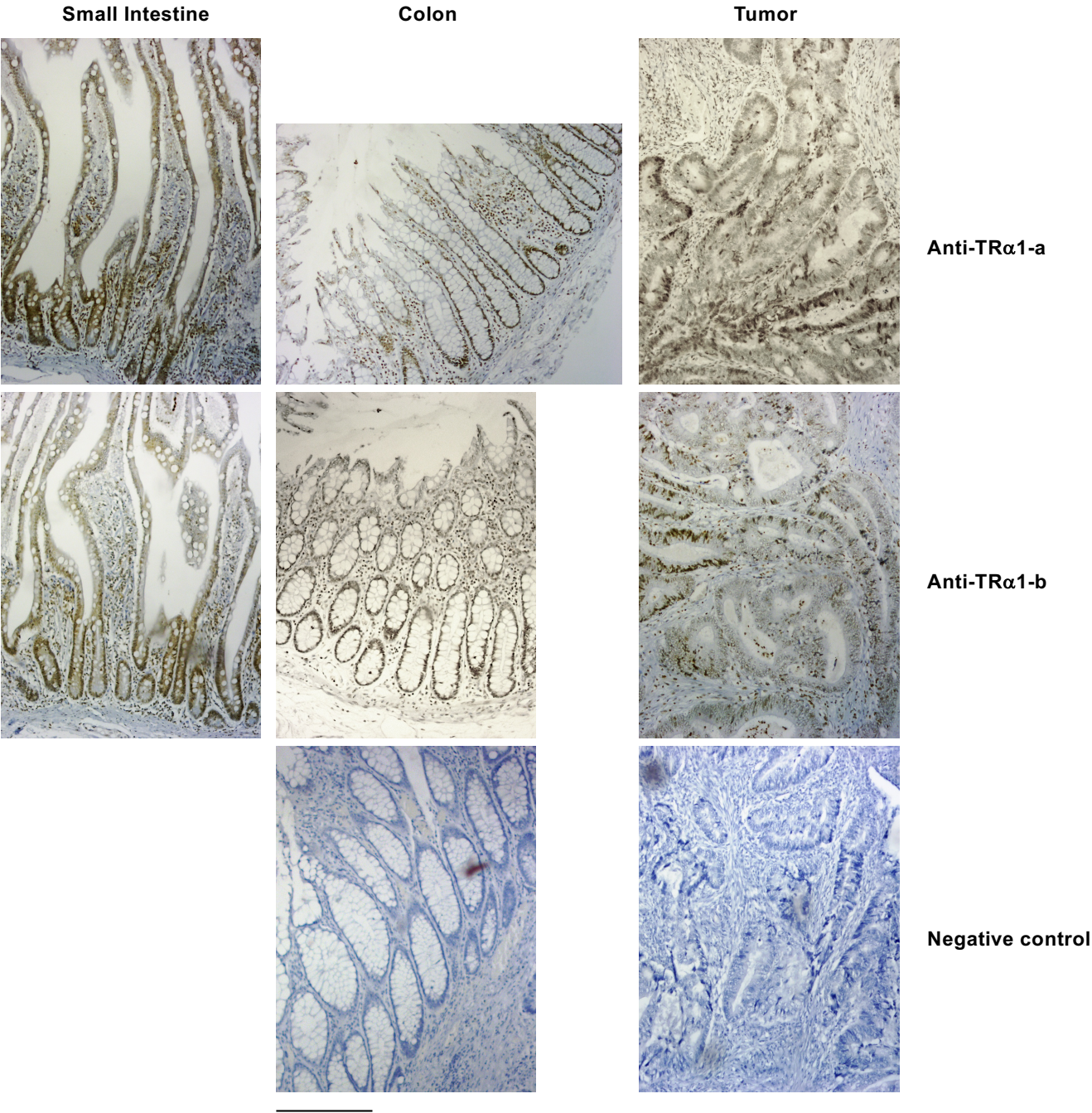

Supplement: Supplementary file 2 — Fig. S2. Setup conditions for TRα1 IHC in human tissue sections. [file MOL2-16-3975-s009.pdf]

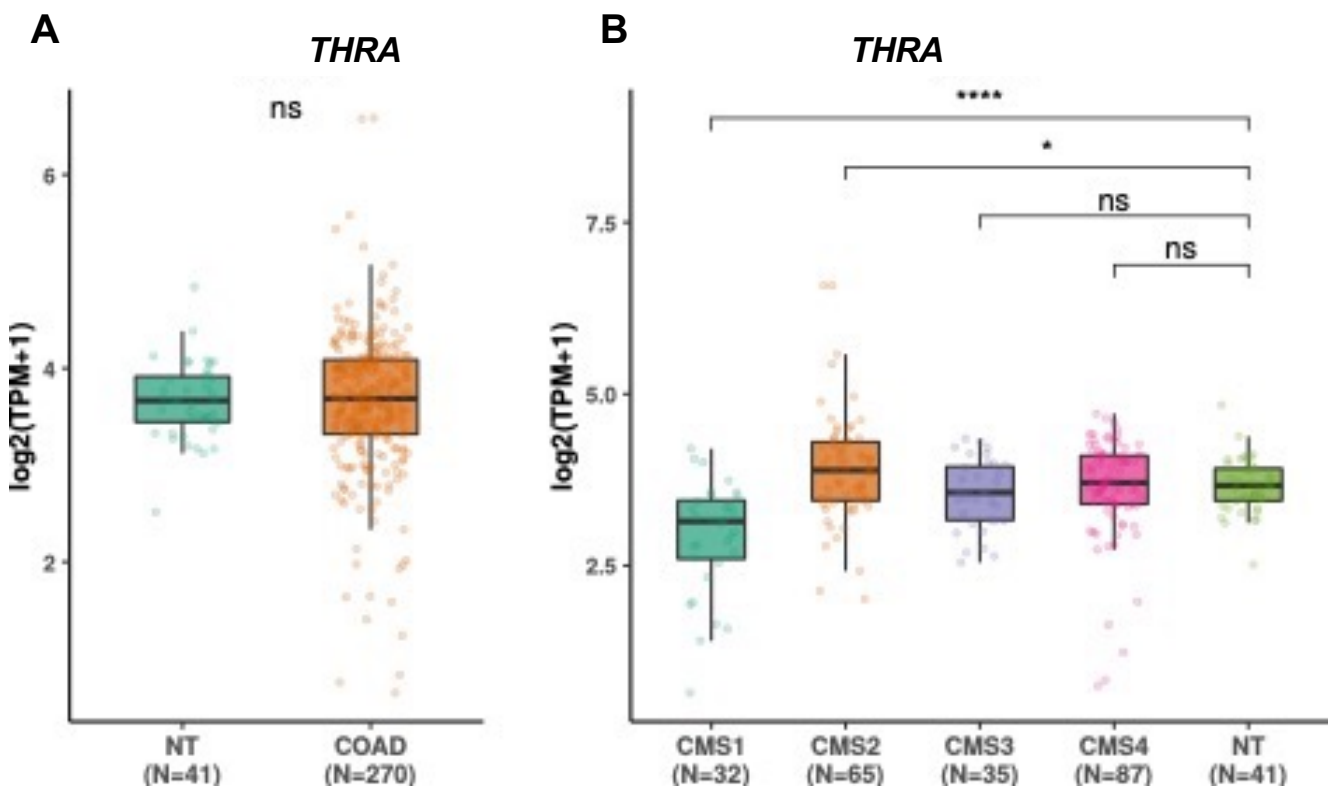

Supplement: Supplementary file 3 — Fig. S3. Analysis of THRA expression in a human colorectal cancer cohort. [file MOL2-16-3975-s011.pdf]

A

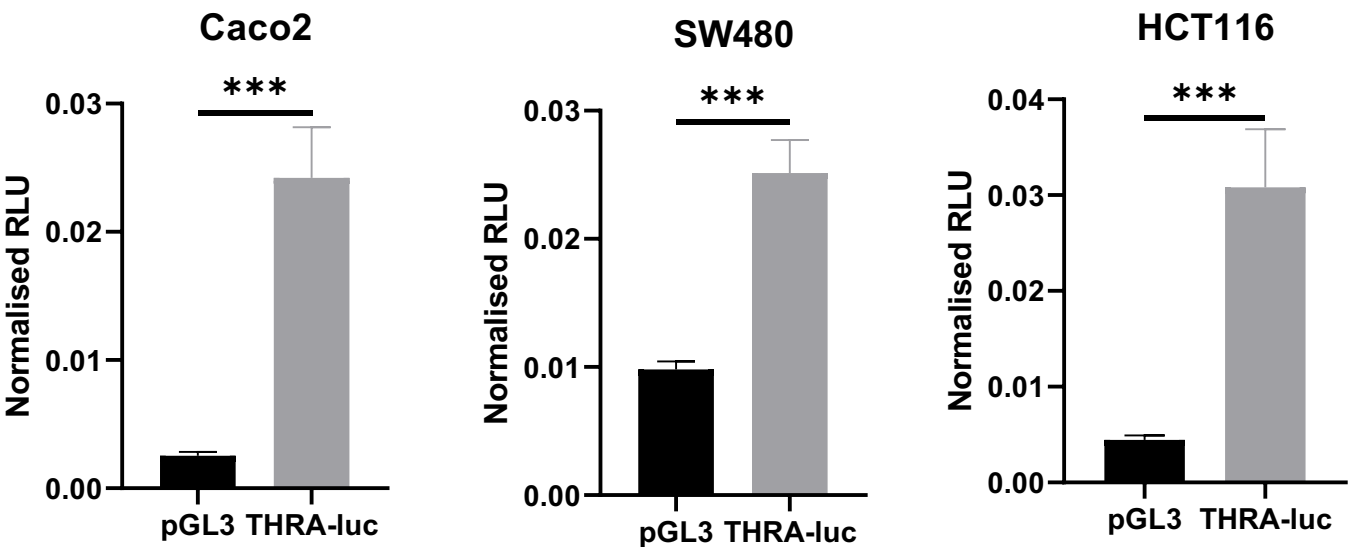

B

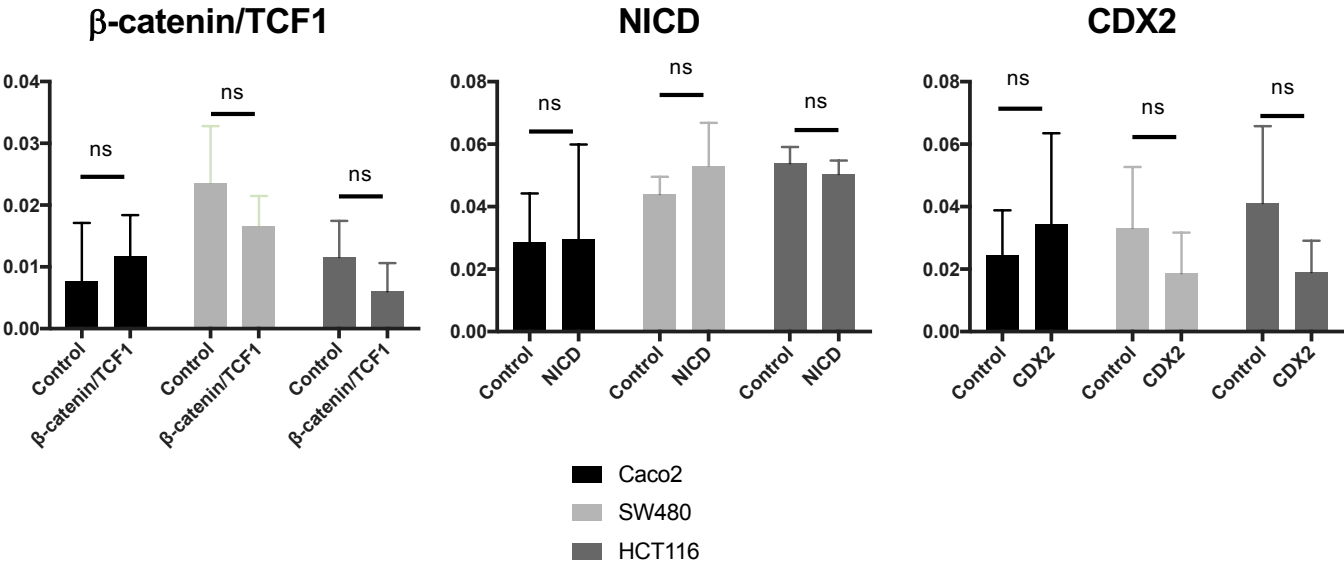

Supplement: Supplementary file 5 — Fig. S5. Analyses of the pGL3‐basic vector. [file MOL2-16-3975-s014.pdf]

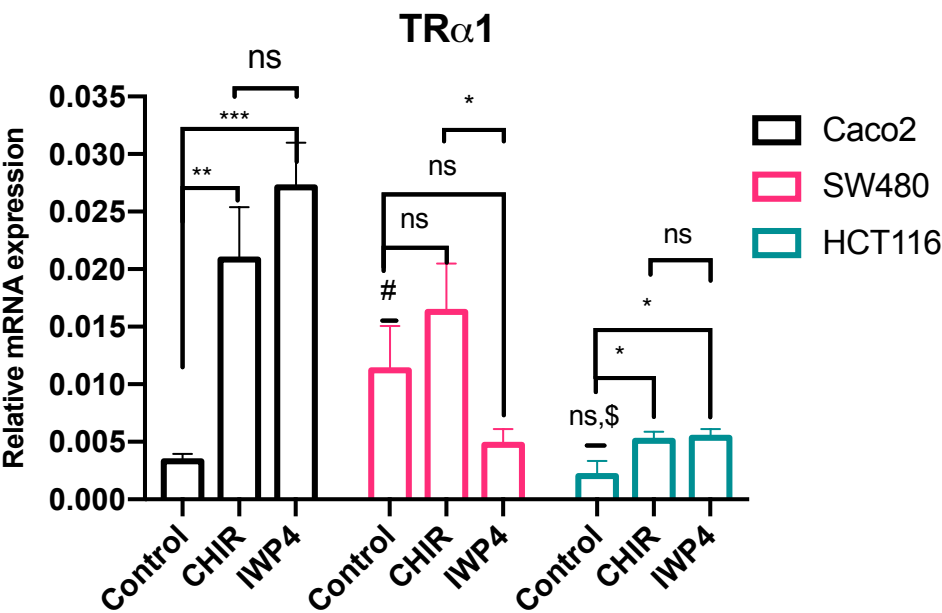

Supplement: Supplementary file 6 — Fig. S6. Effect of the Wnt agonist and antagonist on endogenous TRα1 expression. [file MOL2-16-3975-s004.pdf]

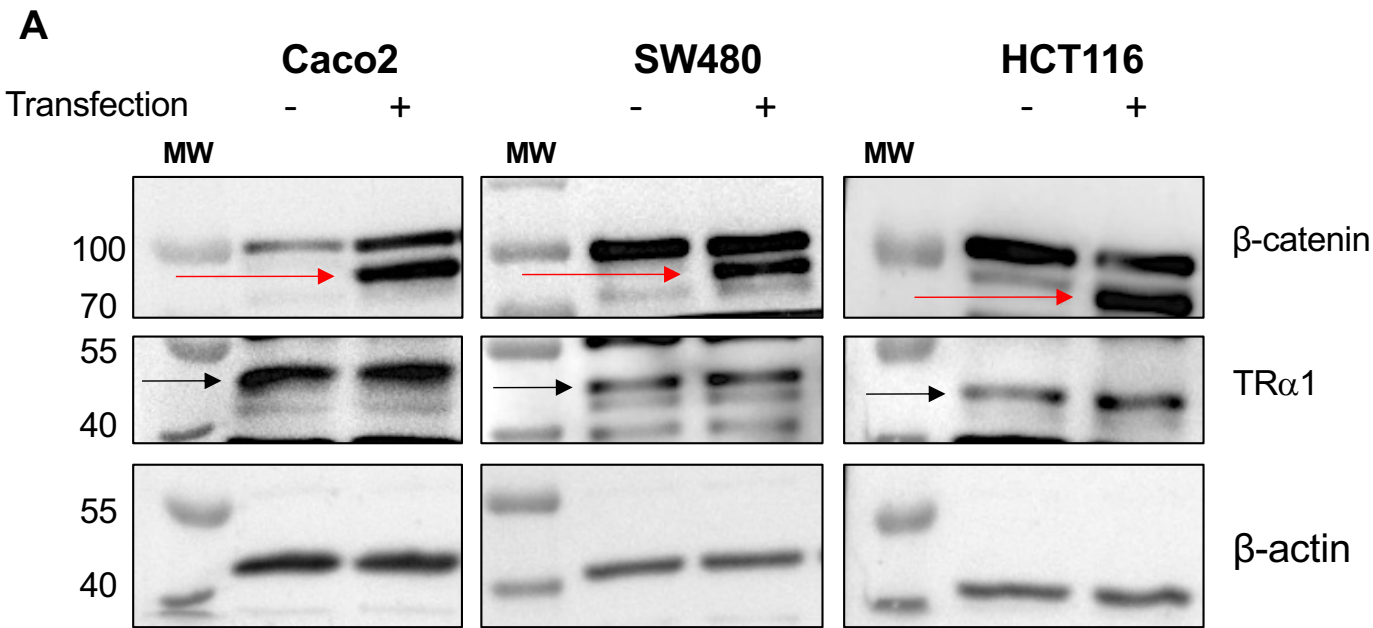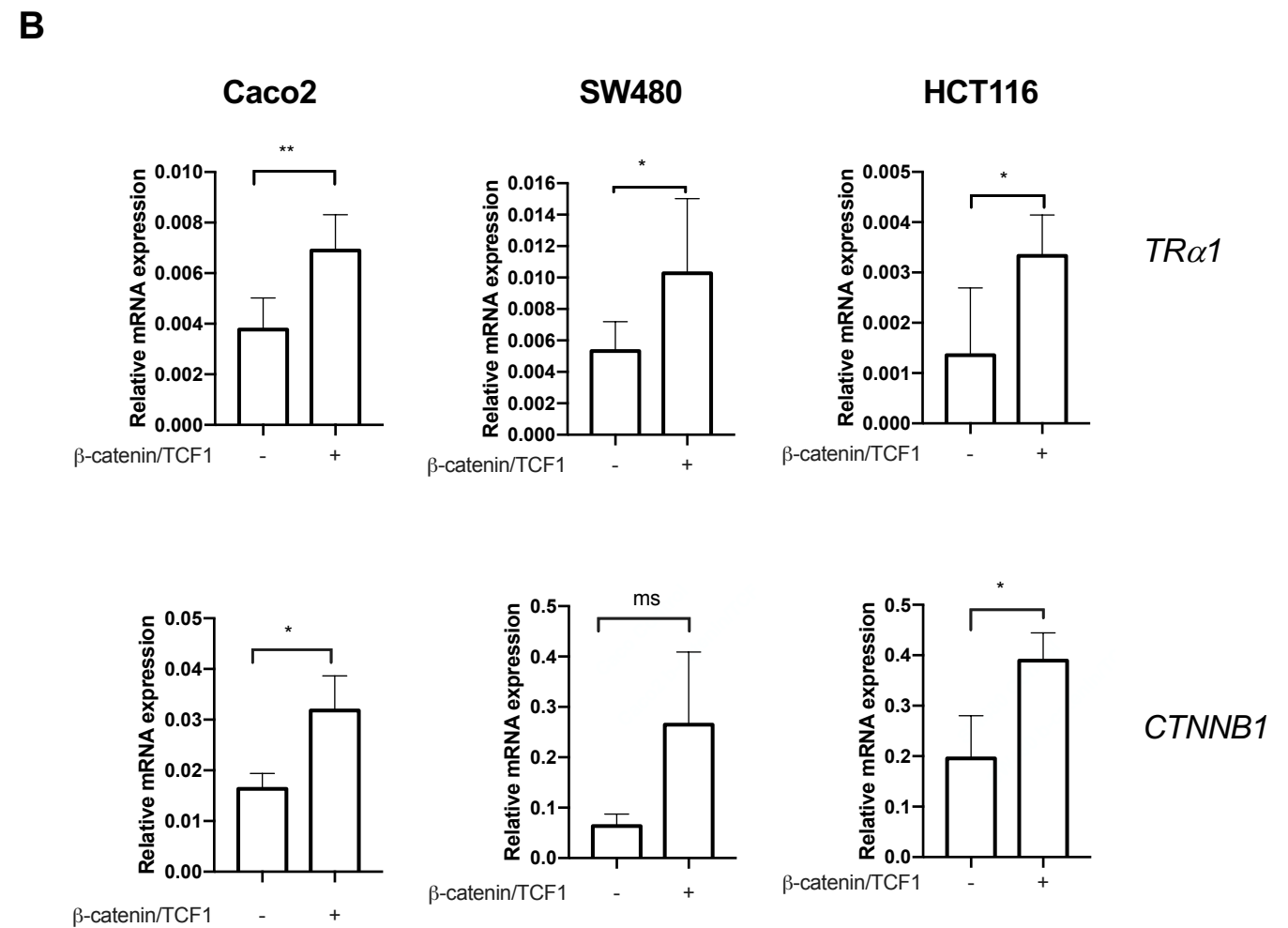

Supplement: Supplementary file 7 — Fig. S7. Effect of β‐catenin/TCF transfection on endogenous TRα1 expression. [file MOL2-16-3975-s013.pdf]

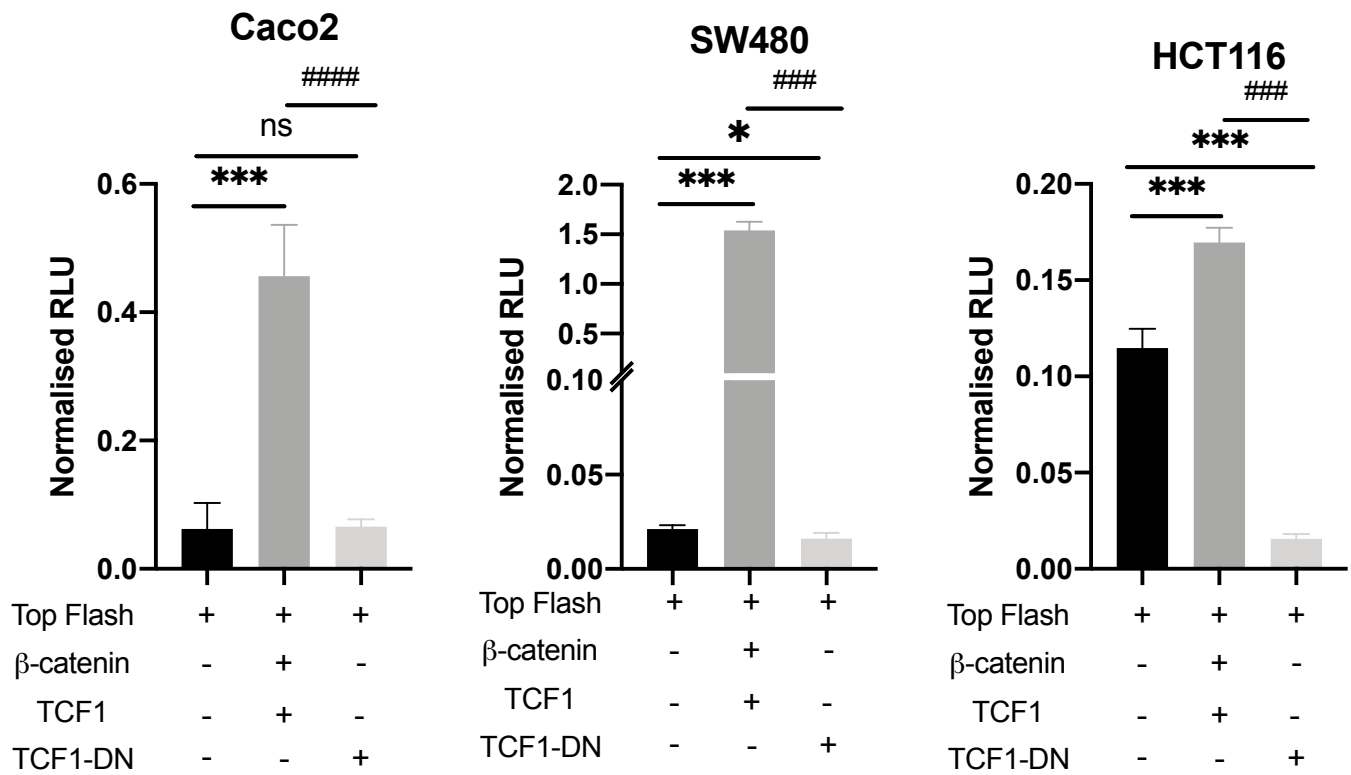

Supplement: Supplementary file 8 — Fig. S8. TopFlash activity is affected in the presence of TCF1‐DN. [file MOL2-16-3975-s001.pdf]

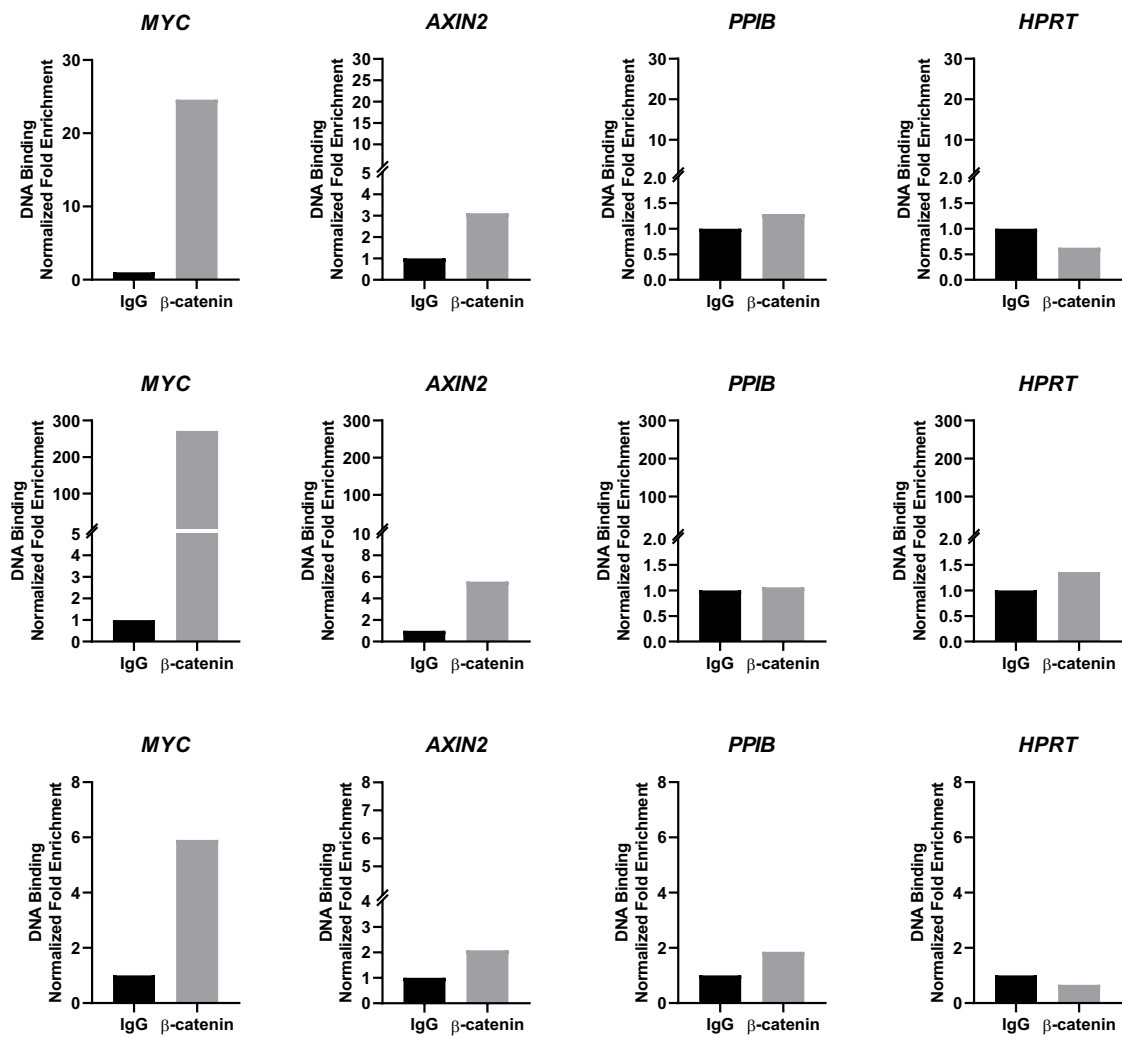

Supplement: Supplementary file 9 — Fig. S9. Chromatin occupancy of β‐catenin in the AXIN2 and MYC promoters. [file MOL2-16-3975-s002.pdf]

Giolito et al, Figure S10

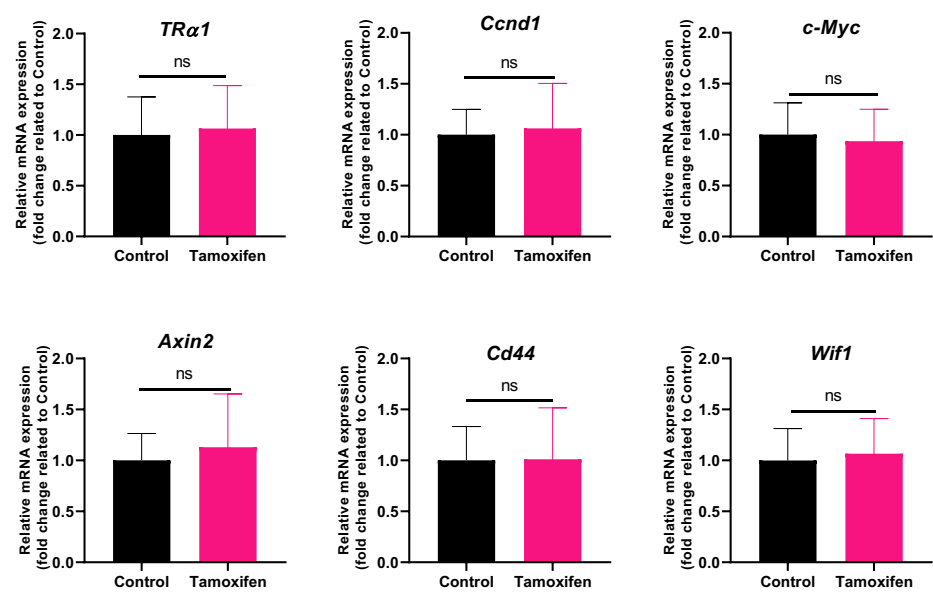

Supplement: Supplementary file 10 — Fig. S10. Complementary analysis on mouse enteroids. [file MOL2-16-3975-s008.pdf]
