# Supplementary material for: Regulation of the THRA gene, encoding the thyroid hormone nuclear receptor TRα1, in intestinal lesions
Source: Mol Oncol. 2022 Oct 10;16(22):3975–93. doi: 10.1002/1878-0261.13298 (PMC9718118; doi:10.1002/1878-0261.13298)

Giolito et al, Figure S4

A

	Microsat. status	*APC*	*CTNNB1*	*TP53*	*KRAS*	*BRAF*	*PI3KCA*	*PTEN*
Caco2	MSS	Mut	Mut	p.E204X	WT	WT	WT	WT
SW480	MSS	Mut	WT	p.R273H; p.P309s	p.G12V	WT	WT	WT
HCT116	MSI	WT	Mut	WT	p.G13D	WT	p.H1047R	WT

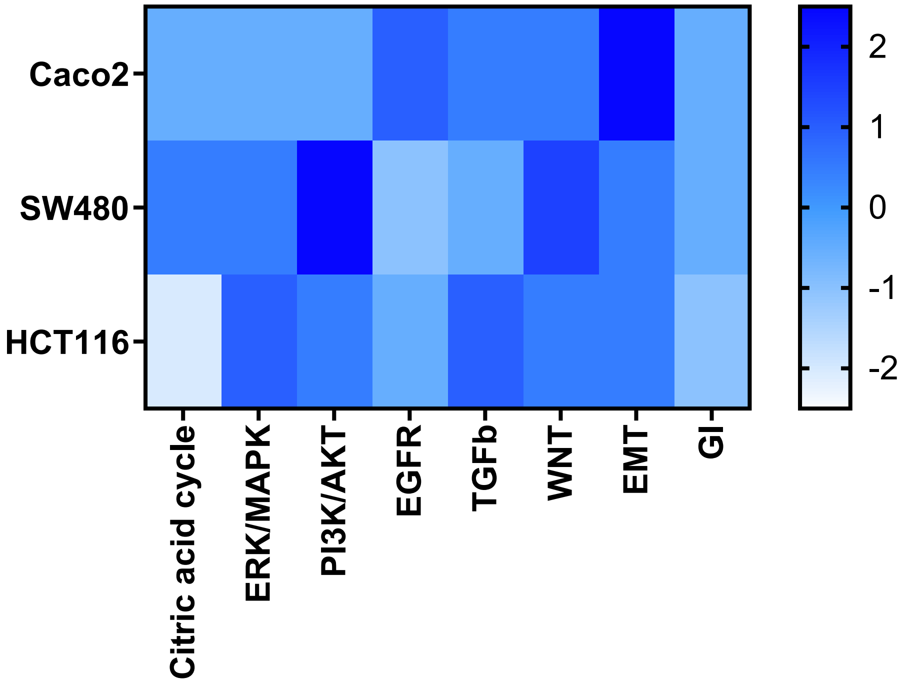

B

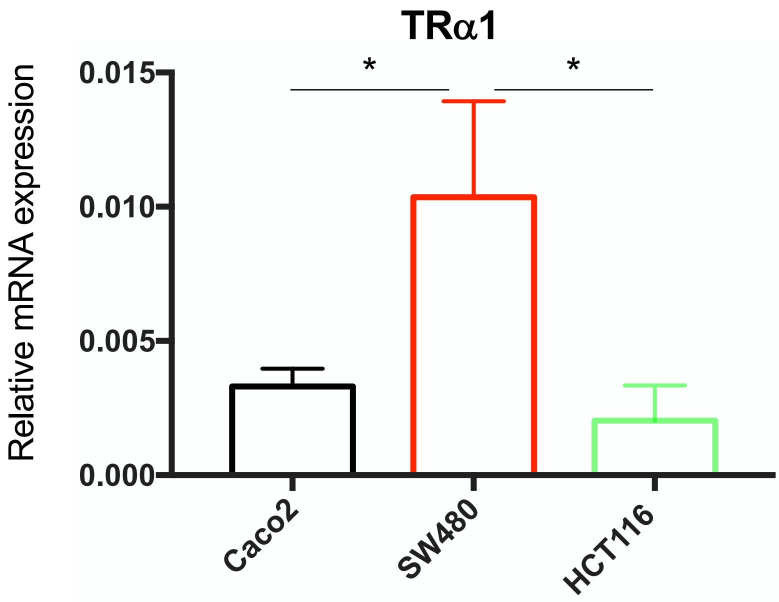

Supplement: Supplementary file 4 — Fig. S4. Characteristics of individual cell lines at multiple molecular levels. [file MOL2-16-3975-s007.pdf]
