# Supplementary material for: Regulation of the THRA gene, encoding the thyroid hormone nuclear receptor TRα1, in intestinal lesions
Source: Mol Oncol. 2022 Oct 10;16(22):3975–93. doi: 10.1002/1878-0261.13298 (PMC9718118; doi:10.1002/1878-0261.13298)
Supplement: Supplementary file 11 — Table S1. List of primers. [file MOL2-16-3975-s005.pdf]

Table S1. List of primers

**Primers used for RT-qPCR**		
**Gene**	**Sequence**	**Product length**
Axin2	F: ATG CTA GGC GGA ATG AAG ATG	250
	R: GGA GAC AAC GCT GTT GTT CTC	
Ccnd1	F: CAG AGG CGG ATG AGA ACA AGT	180
	R: GCG GTA GCA GGA GAG GAA G	
Cd44	F: GAA TGT AAC CTG CCG CTA	267
	R: GGA GGT GTT GGA CGT GAC	
Hprt	F: GCT GGT GAA AAG GAC CTC T	240
	R: CAC AGG ACT AGA ACA CCT GC	
Myc	F: GTT GGA AGA GCC GTG TGT G	129
	R: CGC TGA TGT TGG GTC AGT C	
Ppib	F: CAC CAA TGG CTC ACA GTT CTT	156
	R: ATG ACA TCC TTC AGT GGC TTG	
TRα1	F: TGC CTT TAA CCT GGA TGA CAC	240
	R: TCG ACT TTC ATG TGG AGG AAG	
Wif1	F: ATG TAT TTG CCC TCC TGG ACT	135
	R: GAG CAC AGG TCT CCT TGG TAA	
CTNNB1	F: TGC CAA GTG GGT GGT ATA GAG	126
	R: CCA TCT CTG CTT CTT GGT GTC	
**Primers used for ChIP-qPCR**		
THRA-1	F: GTG GTC TCA AAA TCC TGA CCT	154
	R: TGC TCT CCC AGA TTT ATC CTC	
THRA-2	F: AGA ATT GCT TGA ACC CAG GAG	184
	R: TGT CCC TGT TGA GAG GAA ATG	
AXIN2	F: CTG GAG CCG GCT GCG CTT TGA TAA	98
	R: CGG CCC CGA AAT CCA TCG CTC TGA	
MYC	F: GTG AAT ACA CGT TTG CGG GTT AC	304
	R: AGA GAC CCT TGT GAA AAA AAC CG	
HPRT	F: CCA AAG ATG GTC AAG GTC GC	250
	R: CTG CTG ACA AAG ATT CAC TGG	
PPIB	F: ATG ATC CAG GGC GGA GAC TT	102
	R: GCC CGT AGT GCT TCA GTT TG	
**Primers used to analyze Apc gene deletion**		
$Apc\Delta 14^{fl}$	F: GTT CTG TAT CAT GGA AAG ATA GGT GGT C	800
	R: GAG TAC GGG GTC TCT GTC TCA GTG AA	
