# Supplementary material for: Regulation of the THRA gene, encoding the thyroid hormone nuclear receptor TRα1, in intestinal lesions
Source: Mol Oncol. 2022 Oct 10;16(22):3975–93. doi: 10.1002/1878-0261.13298 (PMC9718118; doi:10.1002/1878-0261.13298)
Supplement: Supplementary file 12 — Table S2. List of antibodies. [file MOL2-16-3975-s012.pdf]

Table S2. List of antibodies

Antibodies used for Western Blot				
**Protein**	**Brand**	**Reference**	**Species**	**Dilution**
β-actin	Sigma	A5441	Mouse	1/10000
β-catenin	Santa Cruz Biotechnology	sc-7963	Mouse	1/500
β-catenin	BD Transduction	Clone 14	Mouse	1/500
TRα1	Abcam	ab53729	Rabbit	1/500
Anti-mouse IgG HRP conjugate	Promega	W402B	Goat	1/5000
Anti-rabbit IgG HRP conjugate	Promega	W401B	Goat	1/5000
