# Supplementary material for: Regulation of the THRA gene, encoding the thyroid hormone nuclear receptor TRα1, in intestinal lesions
Source: Mol Oncol. 2022 Oct 10;16(22):3975–93. doi: 10.1002/1878-0261.13298 (PMC9718118; doi:10.1002/1878-0261.13298)
Supplement: Supplementary file 13 — Table S3. TMA analysis. [file MOL2-16-3975-s003.pdf]

Table S3. TMA analysis

TMA Map Location	Tissue of (Origin/Finding)	Appearance	Sample Pathology from Cytomyx Pathology Verification	Case Diagnosis from Donor Institution Pathology Report	Tumor Grade	TNM	Stage Grouping	TRα1 IHC			
								Tumor	Normal	Stroma	Immune
E3	Colon: right / Colon: right	Tumor	Adenocarcinoma of colon	Adenocarcinoma of colon	AJCC G1: Well differentiated	pT2pN0pMX	I	no tumor	+/-	+	NI
E5	Colon: left / Colon: left	Tumor	Adenocarcinoma of colon	Adenocarcinoma of colon	AJCC G2: Moderately differentiated	pT2pN0pMX	I	++	NP	++	NI
H2	Colon: right / Colon: right	Tumor	Adenocarcinoma of colon	Adenocarcinoma of colon	AJCC G2: Moderately differentiated	pT2pN0pMX	I	+	NP	+/-	NI
B2	Colon: sigmoid / Colon: sigmoid	Tumor	Adenocarcinoma of colon	Adenocarcinoma of colon	AJCC G1: Well differentiated	pT3pN0pMX	IIA	++	NP	++	NI
C2	Colon: right / Colon: right	Tumor	Adenocarcinoma of colon, mucinous	Adenocarcinoma of colon, mucinous	Not Reported	pT3pN0pMX	IIA	-	NP	+/-	++
C4	Colon: right / Colon: right	Tumor	Adenocarcinoma of colon	Adenocarcinoma of colon	AJCC G2: Moderately differentiated	pT3pN0pMX	IIA	+	NP	+	++
D1	Rectum / Rectum	Tumor	Adenocarcinoma of rectum	Adenocarcinoma of rectum	AJCC G2: Moderately differentiated	pT3pN0pMX	IIA	+/-	NP	+	++
D5	Cecum / Cecum	Tumor	Adenocarcinoma of colon	Adenocarcinoma of colon	AJCC G1: Well differentiated	pT3pN0pMX	IIA	+/-,+,++	NP	++	NI
E1	Cecum / Cecum	Tumor	Adenocarcinoma of colon	Adenocarcinoma of colon	Not Reported	pT3pN0pMX	IIA	Section absent			
E4	Colon: right / Colon: right	Tumor	Adenocarcinoma of colon	Adenocarcinoma of colon	AJCC G2: Moderately differentiated	pT3pN0pMX	IIA	++	NP	++	++
F3	Colon: left / Colon: left	Tumor	Adenocarcinoma of colon	Adenocarcinoma of colon	AJCC G2: Moderately differentiated	pT3pN0pM0	IIA	+/-,+,++	NP	++	NI
F4	Colon: hepatic flexure / Colon: hepatic flexure	Tumor	Adenocarcinoma of colon	Adenocarcinoma of colon	AJCC G2: Moderately differentiated	pT3pN0pMX	IIA	++	NP	++	NI
H3	Colon: transverse / Colon: transverse	Tumor	Adenocarcinoma of colon	Adenocarcinoma of colon	AJCC G2: Moderately differentiated	pT3pN0pMX	IIA	+/-,+,++	NP	+	NI
G5	Colon: sigmoid / Colon: sigmoid	Tumor	Adenocarcinoma of colon	Adenocarcinoma of colon	AJCC G1: Well differentiated	pT4pN0pMX	IIB	+/-	NP	+	NI
H5	Colon: sigmoid / Colon: sigmoid	Tumor	Adenocarcinoma of colon	Adenocarcinoma of colon	AJCC G2: Moderately differentiated	pT4pN0pMX	IIB	-	NP	+	+
H1	Colon / Lymph node	Tumor	Adenocarcinoma of colon, metastatic	Adenocarcinoma of colon, metastatic	Not Reported	pTXpN1pMX	IIIA	+	NP	++	++
B1	Colon: sigmoid / Colon: sigmoid	Tumor	Adenocarcinoma of colon	Adenocarcinoma of colon	AJCC G4: Undifferentiated	pT3pN1pMX	IIIB	+	NP	+	NI
C5	Cecum / Cecum	Tumor	Adenocarcinoma of colon	Adenocarcinoma of colon	AJCC G1: Well differentiated	pT3pN1pMX	IIIB	+	NP	+/-	++
E2	Colon: right / Colon: right	Tumor	Adenocarcinoma of colon	Adenocarcinoma of colon	AJCC G2: Moderately differentiated	pT3pN1pMX	IIIB	++	NP	+	NI
F1	Colon / Colon	Tumor	Adenocarcinoma of colon	Adenocarcinoma of colon	AJCC G2: Moderately differentiated	pT3pN1pMX	IIIB	-	NP	+/-	NI
F5	Colon: transverse / Colon: transverse	Tumor	Adenocarcinoma of colon, mucinous	Adenocarcinoma of colon	AJCC G3: Poorly differentiated	pT3pN1pMX	IIIB	Section too damaged			
G4	Cecum / Cecum	Tumor	Adenocarcinoma of colon	Adenocarcinoma of colon	AJCC G3: Poorly differentiated	pT3pN1pMX	IIIB	+/-,+,++	NP	++	NI
C1	Colon: rectosigmoid / Colon: rectosigmoid	Tumor	Adenocarcinoma of colon	Adenocarcinoma of colon	AJCC G2: Moderately differentiated	pT3pN2pMX	IIIC	++	NP	+	NI
D3	Colon: rectosigmoid / Colon: rectosigmoid	Tumor	Adenocarcinoma of colon	Adenocarcinoma of colon	AJCC G2: Moderately differentiated	pT3pN2pMX	IIIC	no tumor	+/-	+/-	++
F2	Colon: left / Colon: left	Tumor	Adenocarcinoma of colon	Adenocarcinoma of colon	Not Reported	pT3pN2pMX	IIIC	+	NP	++	NI
G1	Colon: left / Colon: left	Tumor	Adenocarcinoma of colon	Adenocarcinoma of colon	AJCC G1: Well differentiated	pT4pN2pMX	IIIC	++	NP		NI
G2	Colon: right / Colon: right	Tumor	Adenocarcinoma of colon	Adenocarcinoma of colon	AJCC G1: Well differentiated	pT3pN2pMX	IIIC	++	NP	+	++
G3	Cecum / Cecum	Tumor	Adenocarcinoma of colon, mucinous	Adenocarcinoma of colon, mucinous	AJCC G2: Moderately differentiated	pT3pN2pMX	IIIC	+	NP	+	NI
H4	Cecum / Cecum	Tumor	Adenocarcinoma of colon	Adenocarcinoma of colon	AJCC G1: Well differentiated	pT4pN2pMX	IIIC	++	NP	++	NI
A1	Colon / Liver	Tumor	Adenocarcinoma of colon, metastatic	Adenocarcinoma of colon, metastatic	AJCC G2: Moderately differentiated	pTXpNXpM1	IV	Section absent			
A2	Colon / Liver	Tumor	Adenocarcinoma of colon, metastatic	Adenocarcinoma of colon, metastatic	Not Reported	pTXpNXpM1	IV	Section absent			
A3	Colon: left / Colon: left	Tumor	Adenocarcinoma of colon	Adenocarcinoma of colon	AJCC G2: Moderately differentiated	pT3pN1pM1	IV	+/-,+,++	NP	-	NI
A4	Colon / Liver	Tumor	Adenocarcinoma of colon, metastatic	Adenocarcinoma of colon, metastatic	AJCC G2: Moderately differentiated	pTXpNXpM1	IV	+	NP	+	NI
A5	Colon / Liver	Tumor	Adenocarcinoma of colon, metastatic	Adenocarcinoma of colon, metastatic	Not Reported	pTXpNXpM1	IV	+	NP	+	NI
B3	Colon / Liver	Tumor	Adenocarcinoma of colon, metastatic	Adenocarcinoma of colon, metastatic	Not Reported	pTXpNXpM1	IV	+/-	NP	+	NI
B4	Colon / Liver	Tumor	Adenocarcinoma of colon, metastatic	Adenocarcinoma of colon, metastatic	Not Reported	pTXpNXpM1	IV	+	NP	++	NI
B5	Colon / Liver	Tumor	Adenocarcinoma of colon, metastatic	Adenocarcinoma of colon, metastatic	Not Reported	pTXpNXpM1	IV	-,+/-	NP	-	NI
C3	Colon / Lung	Tumor	Adenocarcinoma of colon, metastatic	Adenocarcinoma of colon, metastatic	Not Reported	pTXpNXpM1	IV	Section absent			
D2	Colon: right / Colon: right	Tumor	Adenocarcinoma of colon	Adenocarcinoma of colon	AJCC G1: Well differentiated	pT3pN0pM1	IV	++	NP	++	NI
D4	Colon / Liver	Tumor	Adenocarcinoma, metastatic, consistent with colon primary	Adenocarcinoma of colon, metastatic	AJCC G2: Moderately differentiated	pTXpNXpM1	IV	Section absent			
I1	Colon / Colon	Normal	Within normal limits	Adenocarcinoma of colon, metastatic	Not Reported	pTXpN1pMX	IIIA	NP	+/-	+/-	NI
I2	Colon: right / Colon: right	Normal	Within normal limits	Adenocarcinoma of colon	AJCC G2: Moderately differentiated	pT2pN0pMX	I	Section absent			
I3	Colon: transverse / Colon: transverse	Normal	Within normal limits	Adenocarcinoma of colon	AJCC G2: Moderately differentiated	pT3pN0pMX	IIA	NP	+/-	+/-	NI
I4	Colon / Colon	Normal	Within normal limits	Adenocarcinoma of colon	AJCC G1: Well differentiated	pT4pN2pMX	IIIC	NP	+/-	+/-	NI
I5	Colon: sigmoid / Colon: sigmoid	Normal	Within normal limits	Adenocarcinoma of colon	AJCC G2: Moderately differentiated	pT4pN0pMX	IIB	Section absent			

NI: Not Identified
NP: Not Present
