# Supplementary material for: Regulation of the THRA gene, encoding the thyroid hormone nuclear receptor TRα1, in intestinal lesions
Source: Mol Oncol. 2022 Oct 10;16(22):3975–93. doi: 10.1002/1878-0261.13298 (PMC9718118; doi:10.1002/1878-0261.13298)
Supplement: Supplementary file 14 [file MOL2-16-3975-s006.pdf]

Supplementary Figure Legends

Figure S1. Schematic representation of the *THRA*-luc constructs. A) *THRA*-luc promoter containing 3238 bp upstream of the transcription starting site of the *THRA* gene cloned into the pGL3-basic vector. **B-D)** *THRA*-mut-luc vectors, including TCF7L2-1-mut (B) containing a CT>GC mutation at 816 bp, TCF7L2-2-mut (C) containing a CT>GC mutation at position 2270 bp and TCF7L2-dmut (D) with mutations in both sites.

Figure S2. Setup conditions for TRα1 IHC in human tissue sections. Two antibodies against TRα1 were used to determine the conditions that produced better efficacy for application in automated TMA analysis. Lab-made anti-TRα1 [14] (dil. 1:100) and commercial anti-TRα1 (ab53729, dil. 1:50), indicated respectively TRα1-a and TRα1-b, were applied to human paraffin sections of the small intestine and colon and colon tumors. In the negative control condition, primary antibody was omitted. Note that the two antibodies produced similar results, with TRα1 expressed in the proliferative/stem cell zone in both the normal small intestine and colon and in a heterogeneous pattern in tumors. Because of the clearer labeling obtained with the commercial antibody, we used it in TMA IHC. Scale bar: 25 µm.

Figure S3. Analysis of *THRA* expression in a human colorectal cancer cohort. *THRA* gene expression levels were evaluated in the TGCA Colon Adenocarcinoma (COAD) cohort. **A)** *THRA* mRNA levels in normal tissue (NT) *vs.* cancer samples. **B)** *THRA* mRNA expression levels according to the four consensus molecular subtypes (CMS) of CRC. NS= nonsignificant, *: P<0.05, and ****: P<0.00001 by the Wilcoxon test.

Figure S4. Characteristics of individual cell lines at multiple molecular levels. A) The table shows the microsatellite status and different common CRC mutations in Caco2, SW480 and HCT116 human adenocarcinoma cell lines. The heat map shows standardized single-sample gene set expression enrichment scores for the eight selected pathways indicated at the bottom in the three cell lines, as adapted from [54]. MSS: microsatellite stable, MSI: microsatellite instable, mut: mutated, WT: wild-type, EMT: epithelium-mesenchyme transition, GI: gastrointestinal. **B)** TRα1 mRNA levels in the three cell lines analyzed by RT-qPCR. The results are representative of at least two independent experiments. Histograms represent the mean ± SD (n=6); *PPIB* was used as a reference gene. *: P<0.05 based on unpaired, two-tailed Student's t test.

Figure S5. Analyses of the pGL3-basic vector. A) Analysis of *THRA*-dependent luciferase activity in Caco2 (left), SW480 (center) and HCT116 (right) cells compared to the original vector used for the generation of the THRA-luc construct. The graphs show the mean ± SD of the normalized relative luciferase units (RLUs) from at least two independent experiments, each of which were conducted using six replicates. ***P<0.001 by unpaired, two-tailed Student's t test. **B-D)** Lack of effect of the Wnt (B) and Notch (C) effectors or of CDX2 (D) on pGL3-basic vector.

Figure S6. Effect of the Wnt agonist and antagonist on endogenous TRα1 expression. TRα1 mRNA levels in the three cell lines analyzed by RT-qPCR under the indicated conditions. The results are representative of at least two independent experiments. Histograms represent the mean ± SD (n=6); *PPIB* was used as a reference gene. NS: not significant, *: P<0.05, **: P<0.01 comparing the different conditions in each cell line #: P<0.05 and $: P<0.05 comparing Caco2 control *vs.* SW480 control or SW480 control *vs.* HCT116. Statistical analyses were performed using an unpaired, two-tailed Student's t test.

Figure S7. Effect of β-catenin/TCF transfection on endogenous TRα1 expression. A) WB analysis performed on protein extracts from the three cell lines in control (-) or β-catenin/TCF transfection conditions (+). β-catenin was analyzed to confirm the transfection efficacy. The results are representative of two independent experiments. The red arrow indicates the truncated form of the transfected β-catenin. The black arrow points to the specific TRα1 band; lower bands represent nonspecific signals. MW: molecular weight marker. **B)** TRα1 and *CTNNB1* mRNA levels in the three cell lines analyzed by RT-qPCR under the indicated conditions. The results are representative of two independent experiments. Histograms represent the mean ± SD (n=6); *PPIB* was used as a reference gene. NS: not significant, *: P<0.05, **: P<0.01 by comparing the transfected *vs.* the control condition using an unpaired, two-tailed Student's t test.

Figure S8. TopFlash activity is affected in the presence of TCF1-DN. TopFlash was used as the positive control for Wnt activity. Caco2 (left panel), SW480 (central panel) and HCT116 (right panel) cells were transfected with TopFlash alone or cotransfected with β-catenin, TCF1 or TCF1-DN, as indicated. Graphs show the mean ± SD of normalized relative luciferase units (RLU) from at least two independent experiments, each conducted in six replicates. NS: nonsignificant, *: P<0.05, and ***: P<0.001 by comparing basal activity with cotransfection conditions; ###: P<0.001 by comparing TopFlash activity when cotransfected with β-catenin/TCF1 or β-catenin/TCF1-DN by unpaired, two-tailed Student's t-test.

Figure S9. Chromatin occupancy of β-catenin in the AXIN2 and MYC promoters. ChIP analysis was performed with chromatin prepared from **A)** Caco2, **B)** SW480 and **C)** HCT116 cells and immunoprecipitated using an anti-β-catenin or IgG (negative control). qPCR was performed using specific primers covering TCF7L2 binding sites within the *AXIN2* and *MYC* promoters (positive controls). Nonspecific binding of β-catenin was evaluated using primers for the *HRPT* and *PPIB* genes (negative controls). The results are representative of two independent experiments. Histograms represent the fold enrichment of specific β-catenin/DNA binding normalized to the input and compared with the IgG condition (=1).

Figure S10. Complementary analysis on mouse enteroids. RT-qPCR analysis for the indicated genes performed using RNA isolated from $Apc^{+/fl}$ enteroids treated with tamoxifen or not treated (control), as indicated. The results are representative of three independent experiments, each conducted on six replicates. Histograms show the mean ± SD (n=6), and data are expressed as

the fold change relative to the control condition (=1). *Ppib* was used as a reference gene. NS: nonsignificant by unpaired, two-tailed Student's t-test.
